# Discriminating Spontaneous From Cigarette Smoke and THS 2.2 Aerosol Exposure-Related Proliferative Lung Lesions in A/J Mice by Using Gene Expression and Mutation Spectrum Data

**DOI:** 10.3389/ftox.2021.634035

**Published:** 2021-03-16

**Authors:** Yang Xiang, Karsta Luettich, Florian Martin, James N. D. Battey, Keyur Trivedi, Laurent Neau, Ee Tsin Wong, Emmanuel Guedj, Remi Dulize, Dariusz Peric, David Bornand, Sonia Ouadi, Nicolas Sierro, Ansgar Büttner, Nikolai V. Ivanov, Patrick Vanscheeuwijck, Julia Hoeng, Manuel C. Peitsch

**Affiliations:** ^1^Philip Morris International R&D, Philip Morris Products S.A., Neuchâtel, Switzerland; ^2^Philip Morris International R&D, Philip Morris International Research Laboratories Pte. Ltd., Singapore, Singapore; ^3^Histovia GmbH, Overath, Germany

**Keywords:** cigarette smoke, heated tobacco product, mouse, lung tumor, gene signature, tumor classification

## Abstract

Mice, especially A/J mice, have been widely employed to elucidate the underlying mechanisms of lung tumor formation and progression and to derive human-relevant modes of action. Cigarette smoke (CS) exposure induces tumors in the lungs; but, non-exposed A/J mice will also develop lung tumors spontaneously with age, which raises the question of discriminating CS-related lung tumors from spontaneous ones. However, the challenge is that spontaneous tumors are histologically indistinguishable from the tumors occurring in CS-exposed mice. We conducted an 18-month inhalation study in A/J mice to assess the impact of lifetime exposure to Tobacco Heating System (THS) 2.2 aerosol relative to exposure to 3R4F cigarette smoke (CS) on toxicity and carcinogenicity endpoints. To tackle the above challenge, a 13-gene gene signature was developed based on an independent A/J mouse CS exposure study, following by a one-class classifier development based on the current study. Identifying gene signature in one data set and building classifier in another data set addresses the feature/gene selection bias which is a well-known problem in literature. Applied to data from this study, this gene signature classifier distinguished tumors in CS-exposed animals from spontaneous tumors. Lung tumors from THS 2.2 aerosol-exposed mice were significantly different from those of CS-exposed mice but not from spontaneous tumors. The signature was also applied to human lung adenocarcinoma gene expression data (from The Cancer Genome Atlas) and discriminated cancers in never-smokers from those in ever-smokers, suggesting translatability of our signature genes from mice to humans. A possible application of this gene signature is to discriminate lung cancer patients who may benefit from specific treatments (i.e., EGFR tyrosine kinase inhibitors). Mutational spectra from a subset of samples were also utilized for tumor classification, yielding similar results. “Landscaping” the molecular features of A/J mouse lung tumors highlighted, for the first time, a number of events that are also known to play a role in human lung tumorigenesis, such as Lrp1b mutation and Ros1 overexpression. This study shows that omics and computational tools provide useful means of tumor classification where histopathological evaluation alone may be unsatisfactory to distinguish between age- and exposure-related lung tumors.

## Introduction

The carcinogenic risk of a chemical is traditionally assessed in 2-year rodent carcinogenicity assays selecting the relevant route of administration for the compound to be tested. Despite concerns regarding the use of large numbers of animals, exposures that are not relevant to humans, and the sometimes poor translatability to human outcomes, not to mention prohibitive costs and time needed (Cohen, 2010; Osimitz et al., 2013), the 2-year bioassay remains a standard for the identification of human cancer hazards. Mice have been employed to elucidate the underlying mechanisms of lung tumor formation and progression and to derive human-relevant modes of action (Meuwissen and Berns, 2005). Different strains of mice display markedly varied sensitivity to lung tumor development (Gordon and Bosland, 2009). For example, mice of the C57Bl/6 strain are quite resistant to tumor induction, while Balb/c mice are considered intermediate in susceptibility. In contrast, the A/J mouse is highly susceptible to lung tumor induction and has been widely used as a screening system in carcinogenicity testing. In this inbred strain, K-ras oncogene activation is associated with an enhanced risk for lung tumor susceptibility (Lin et al., 1998), illustrated by the development of pulmonary adenoma. This suggests that the model, at least in part, reflects molecular events during human lung tumorigenesis. Our previous studies with mainstream cigarette smoke (CS) from the 3R4F reference cigarette showed that chronic exposure was sufficient to elicit a concentration-dependent lung tumor response (Stinn et al., 2013a,b), in line with earlier findings (Curtin, 2004; Witschi et al., 2006). However, the A/J mouse model also has the disadvantage that spontaneous lung tumors arise as the animals age and that these spontaneous tumors are histologically indistinguishable from the tumors occurring in CS-exposed mice (Gordon and Bosland, 2009). We previously explored the molecular characteristics of these 2 tumor types using gene and microRNA (miRNA) expression analysis (Luettich et al., 2014). A 50-gene expression signature was extracted, which separates lung tumors into 2 groups−1 reflecting the gene signature profiles of all tumors in the sham and low total particulate matter (TPM) exposure groups, and 1 comprising the medium and high TPM exposure group tumors. Changes in gene and miRNA expression profiles suggested that tumors from CS-exposed mice were equipped to escape from immune surveillance by dysregulation of humoral immune responses and glycosphingolipid metabolism. Together, these molecular features indicated that lung tumors in exposed mice diverged from those spontaneously arising in aging A/J mice. This resembles observations in lung cancer patients with or without prior smoking history, in whom chronic CS exposure leads to distinct molecular features in lung tumors that are absent in lung tumors from non-smokers [reviewed by Smolle and Pichler (2019)]. The existence of distinct molecular features mentioned above motivated us to develop a gene signature to tackle the challenge, distinguishing tumors in CS-exposed animals from spontaneous tumors, which cannot be handled by histopathological evaluation alone.

We wanted to further explore the molecular differences in proliferative lung lesions from another chronic toxicity/carcinogenicity study in A/J mice, in which animals were not only exposed to CS but also to an aerosol from the Tobacco Heating System (THS) 2.2. Because THS 2.2 aerosol contains significantly lower levels of harmful and potentially harmful constituents than CS (Schaller et al., 2016), including those with known carcinogenic properties [e.g., 1,3-butadiene, benzene, benzo(a)pyrene, 4-(N-nitrosomethylamino)-1-(3-pyridyl)-1-butanone [NNK]], we expected that chronic exposure of animals would result in different lung tumor incidence and multiplicity than CS exposure. We also expected the 2 types of aerosols—CS vs. THS 2.2 aerosol—to have differential effects on the molecular makeup of proliferative lung lesions that could be indicative of their divergence from spontaneous lesions in the lungs of air-exposed A/J mice. There were three types of lung tumors: Spontaneous tumors, 3R4F CS-related tumors, and tumors from THS 2.2 aerosol-exposed mice, as shown in Supplementary Table 1.

## Methods

### Inhalation Study

We conducted a chronic toxicity/carcinogenicity study with the candidate modified risk tobacco product THS 2.2 based on the OECD Test Guideline 453: Combined Chronic Toxicity/Carcinogenicity Studies (OECD, 2018) in A/J mice (Jackson Laboratory, Bar Harbor, ME, USA). The focus of the study was on the OECD endpoints (i.e., the toxicity due to lifetime inhalation of mainstream THS 2.2 aerosol and tumor endpoints relative to the toxicity inherent in the inhalation of mainstream CS from the 3R4F reference cigarette). We also sought to examine the extent of lung inflammation and emphysematous changes and characterize molecular changes in the respiratory tract using a systems toxicology approach. The study design, analytical characterization of selected aerosol constituents in the test atmospheres, biomarkers of exposure in the blood and urine samples of exposed mice, general health conditions of the mice, and histopathological findings, including non-proliferative and proliferative respiratory tract findings, are described in another publication (Wong et al., 2020). Additionally, we report the results of extensive omics analyses of nasal and laryngeal epithelia and the whole lung (Titz et al., 2020).

The THS 2.2 HeatStick, the test item, has been described previously (Smith et al., 2016). 3R4F cigarettes, which were used as the reference, were obtained from the University of Kentucky (2003). THS 2.2 HeatSticks and cigarettes were conditioned in accordance with ISO standard 3402 (ISO3402, 1999) before being used for aerosol generation. Mainstream smoke from 3R4F cigarettes and aerosol from THS 2.2 HeatSticks were generated as previously described (Wong et al., 2016).

In brief, female A/J mice (9–11 weeks old) were whole-body exposed to aerosol from THS 2.2 at 3 test atmosphere concentrations of nicotine [6.7 (Low, L), 13.4 (Medium, M), and 26.8 (High, H) μg nicotine/L test atmosphere] or to 1 concentration of 3R4F CS (13.4 μg nicotine/L test atmosphere) in whole-body inhalation chambers for 6 h per day, 5 days per week. The nicotine concentration in THS (M) matched that in CS; the CS concentration was chosen on the basis of prior data indicating a robust lung tumor response in this mouse strain (Stinn et al., 2013a,b). Necropsies were carried out after 1, 5, 10, and 18 months of exposure. Male mice were exposed either to fresh air (sham) or to the high THS 2.2 aerosol concentration for 15 months. The group design for female mice was in alignment with OECD TG453; two concurrent controls were included (fresh air and cigarette smoke as negative and positive controls, respectively), and the test item aerosol was supplied at the maximum tolerated dose (MTD) based on nicotine toxicity (THS2.2 High) and two additional lower doses at half (THS2.2 Medium) and one quarter (THS2.2 Low) the MTD, respectively. The group design for male mice deviated from OECD TG453 in that they were only exposed to fresh air or THS 2.2 aerosol at the MTD. In line with the 3R principles, specifically the reduction of animal use, the male CS exposure group was omitted, as we previously observed that female mice (and rats) are more sensitive to the toxicological effects of cigarette smoke than their male counterparts and that CS exposure induces similar lung tumor multiplicity in male and female A/J mice (Stinn et al., 2013a,b).

Housing and all procedures involving animals were performed in accordance with the approved Institutional Animal Care and Use Committee (IACUC) protocol in a facility licensed by the Agri-Food & Veterinary Authority of Singapore (AVA) and accredited by the Association for Assessment and Accreditation of Laboratory Animal Care International (AAALAC), where the procedures for care and use of animals for scientific purposes were in accordance with the NACLAR Guidelines (NACLAR 2004). Additional details about the study design, animal husbandry, aerosol generation, animal exposure, and monitoring are provided in the Supplementary Materials and Methods.

### Lung Tissue Collection

Lung tumors in A/J mice begin to develop at around month 5 (Stinn et al., 2013a). Therefore, lungs were collected after 5, 10 and 18 months exposure from female animals (*N* = 8, 10–12, and 10–13, respectively, per treatment group), and at terminal dissection from male animals [*N* = 16 and 5 for the sham and THS (H) groups, respectively].

Animals from each group were necropsied within 16–24 h of the last exposure and subjected to gross pathology examination. Lungs were perfused *in situ* with cold, sterile, calcium- and magnesium-free phosphate-buffered saline (PBS; MilliporeSigma, Singapore). The whole lung with trachea and larynx was then removed from the animal, rinsed with sterile PBS, blotted dry, and placed in a sterile petri dish. The trachea was cannulated using an 18G catheter, and lungs were inflated slowly with 50% (v/v) Tissue-Tek® optimum cutting temperature (OCT) compound (InLab Supplies Pte Ltd, Singapore) in sterile PBS at a rate of ~0.1 mL per 10 s from a syringe. The volume of 50% (v/v) OCT/PBS required to fully inflate a lung was ~1–1.5 mL and dependent on the size of the animal. When each lung lobe was fully inflated, the bronchus leading to each lobe was clamped with forceps, and each lobe was dissected and placed individually into a disposable Tissue-Tek Cryomold® (InLab Supplies Pte Ltd) prefilled with OCT compound. The filled Cryomolds® were placed into isopentane precooled with liquid nitrogen, and frozen tissues were stored at ≤-70°C until further processing.

### Laser-Capture Microdissection

Laser-capture microdissection (LCM) was used to specifically collect lung parenchymal tissue (“parenchyma”) or tissue from each identified proliferative lung lesion (i.e., nodular bronchioalveolar hyperplasia, bronchioalveolar adenoma, and bronchioalveolar adenocarcinoma, collectively referred to here as “lung tumors” for simplicity) under the guidance of the study pathologist. To do so, serial lung cryosections at 20 μm distance were placed, 3 consecutive sections at a time, on sterilized, RNase-free membrane slides (Carl Zeiss Microscopy LLC, Cambridge, UK). Slides were transferred immediately for fixing and staining with 1% (w/v) cresyl violet (Sigma-Aldrich, Buchs, Switzerland). Stained, air-dried sections were then reviewed by the study pathologist, who identified proliferative lung lesions in each section. These lesions were subjected to LCM using the PALM MicroBeam (Carl Zeiss Microscopy LLC). LCM tissue samples were transferred to opaque AdhesiveCap 500 tubes (Carl Zeiss Microscopy LLC) and stored at −80°C until RNA and DNA extraction (generally for <2 weeks). In total, 172 parenchyma and 101 tumor samples were collected for gene expression analysis, and 172 parenchyma and 73 tumor samples were collected for DNA sequencing (Supplementary Table 1).

### Gene Expression Analysis

Sample randomization was performed prior to RNA extraction as a complete block randomization, where the blocking factor was defined by both the type of exposure (study/treatment group) and the dissection time point. The purpose of block randomization is to blind the analysts who conducted RNA extraction and gene expression analysis, and to prevent potential confounding batch effect.

Total RNA was isolated from the LCM tissues using the RNeasy Micro Kit (QIAGEN, Hilden, Germany) following the manufacturer's instructions for QIAcube (QIAGEN) automated extraction. The isolated RNA was subjected to quality control (QC) checks using the Agilent 2100 Bioanalyzer (Agilent Technologies, Santa Clara, CA, USA), and the quantity of the isolated RNA was determined using NanoDrop 1000 spectrophotometers (Thermo Fisher Scientific, Waltham, MA, USA). Because a pre-study optimization phase indicated that the RNA Integrity Number is not reliable in this particular sample type, sample quality was evaluated based on BioAnalyzer traces, and all RNA samples exhibiting typical ribosomal RNA peaks (a sharp peak at 22.5 ± 2.5 s for the alignment, a sharp peak at 42.5 ± 2.5 s corresponding to 18 s ribosomal subunit and a sharp peak at 49.5 ± 2.5 s corresponding to 28 s ribosomal subunit) were processed for the downstream microarray analysis.

Two ng total RNA were processed using the Affymetrix® HT 3′-IVT Pico kit (Thermo Fisher Scientific, Santa Clara, CA, USA). The resulting double stranded cDNA was then hybridized to GeneChip® Mouse Genome 430 2.0 Arrays (Thermo Fisher Scientific) in a GeneChip® Hybridization Oven 645 (Thermo Fisher Scientific) according to the manufacturer's instructions. Arrays were rinsed and stained on a GeneChip® FS450 DX Fluidics Station (Thermo Fisher Scientific) using the Affymetrix® GeneChip® Command Console® Software (AGCC v3.2, protocol FS450_0001). Finally, microarrays were scanned using a GeneChip® Scanner 3000 7G (Thermo Fisher Scientific). Raw images from the scanner were saved as DAT files, which were automatically gridded by the AGCC software to give Affymetrix CEL files.

The raw CEL files were background-corrected, normalized, and summarized using the frozen robust multiarray analysis (Bolstad et al., 2005; Dai et al., 2005). Quality checks, including log-intensities, normalized-unscaled standard error, relative log expression (RLE), median absolute value RLE, and pseudo-images, were performed with the affyPLM package of Bioconductor (Bolstad et al., 2003, 2005). This process led to the exclusion of data from 10 parenchyma and 19 tumor samples because of unsatisfactory quality (Supplementary Table 1). As a consequence, there were only parenchyma but no tumor data in the sham group for month 5 [*N* = 7, 8, 8, 7, and 8 for sham, 3R4F, THS (L), THS (M) and THS (H), respectively, for parenchyma tissue]. Month 10 data included those from 10 to 4 parenchyma and tumor samples from the sham group, 7 and 5 parenchyma and tumor samples from the 3R4F group, 12 and 5 parenchyma and tumor samples from the THS (L) group, 11 and 4 parenchyma and tumor samples from the THS (M) group and 12 parenchyma samples from the THS (H) group. Month 18 data derived from 10 to 5 parenchyma and tumor samples from the sham group, 10 and 16 parenchyma and tumor samples from the 3R4F group, 10 and 9 parenchyma and tumor samples from the THS (L) group, 13 and 12 parenchyma and tumor samples from the THS (M) group and 12 and 9 parenchyma and tumor samples from the THS (H) group. The month 15 samples from the male animals included 16 and 8 parenchyma and tumor samples from the sham group and 5 and 2 parenchyma and tumor samples from the THS (H) group.

### Interaction Analysis

Gene expression data from A/J mouse lung parenchyma (P) and tumor (T) samples from a previous inhalation study (accession number: E-MTAB-1871) were analyzed for interaction effects between tissue type (T or P) and between air/sham and CS exposure using a linear model (Luettich et al., 2014). The RNA expression values of multiple samples were averaged if these multiple samples from the same animal, for parenchyma tissue and tumor tissue, respectively. The genes with significant interaction are those whose levels were differentially affected between the 2 tissue types upon exposure (Supplementary Figure 1). As the tumor sample and parenchyma sample from one animal may be not completely independent, for this study, the interaction model was adapted to consider tumor and parenchyma pairing information and employed to identify interaction effects between tissue type (T or P) and between exposures (CS or THS 2.2 aerosol vs. air). The interaction model in Luettich et al. (2014) cannot be directly used in this study because of this pairing information. The difference of RNA expression values per gene between the tumor and parenchyma samples for every animal, *GxP*, was computed, to remove the possible dependence. Then a statistical model is fitted based on the independent samples, as follows:


(1) $$\begin{array}{r} △{GxP}_{\;i,j}=\beta_{0,i}+\beta_{1,i}\times \;ExposureType_{j}+\varepsilon_{i,j} \end{array}$$

with i = 1, …, p and j = 1, …, n, where p denotes the total number of genes, 17,473; n denotes the number of independent samples (mice) in the above model; ExposureType is 3R4F CS (13.4 ug/l nicotine), or THS 2.2 Low (6.7 μg/l nicotine), or THS 2.2 Med (13.4 μg/l nicotine), or THS 2.2 High (26.8 μg/l nicotine), respectively; β_1, *i*_ and β_0, *i*_ denote the interaction coefficient and the intercept, respectively, for gene *i*; ε_*i, j*_ is the error term. This model was separately applied to the combination of 4 different ExposureType aforementioned and the time points (months 10, 15, and 18), as a single model may not fulfill homoscedasticity conditions.

**Figure 1:**
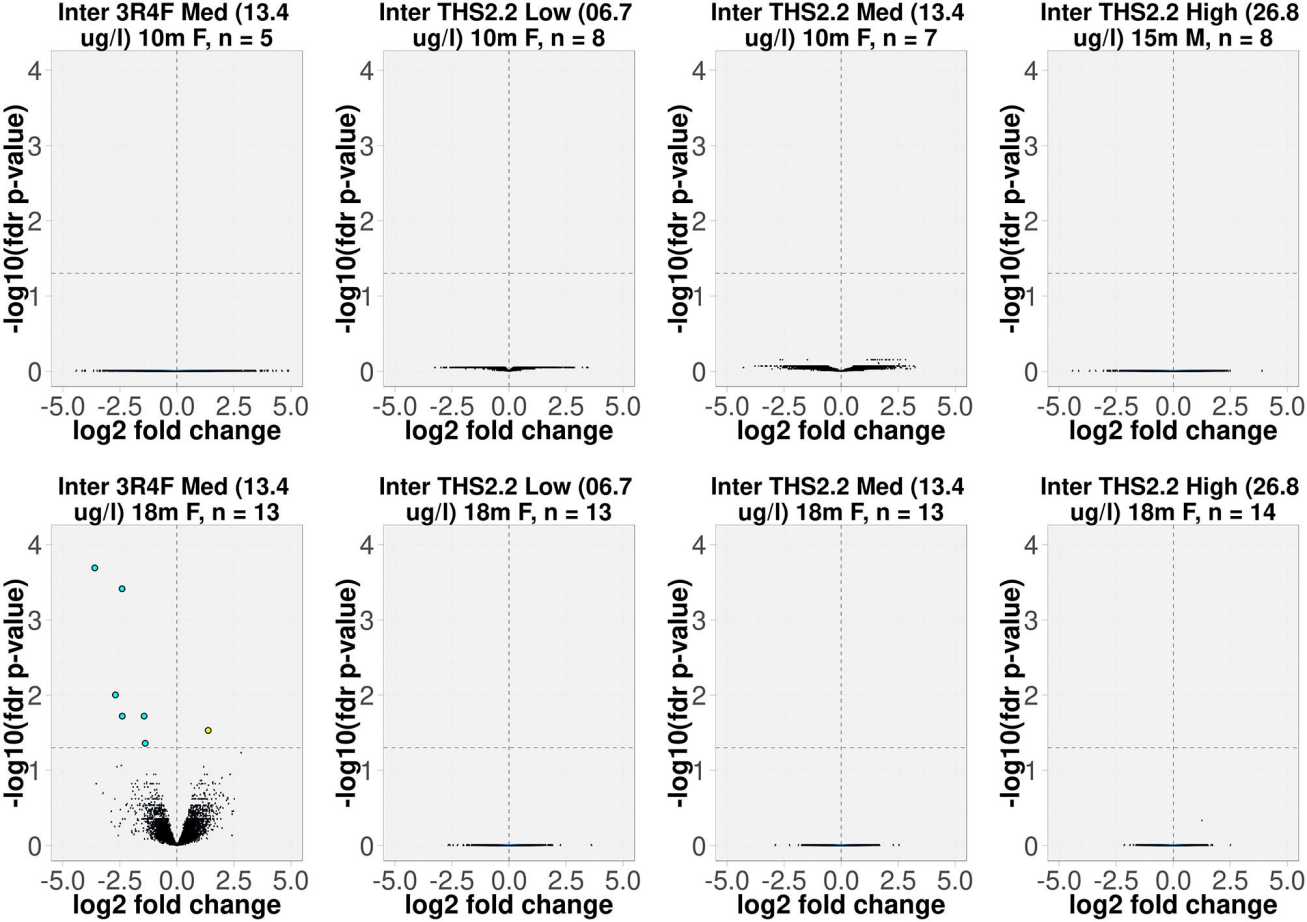
Volcano plots representing the expression profiles of significant interaction terms. The interaction term reflects the changes in gene expression (due to exposure), which were different in tumors compared to the surrounding parenchyma tissues. The interaction value for each gene, denoted as log2 fold change, is plotted on the x-axis, and the statistical significance, proportional to the –log_10_ of the fdr-adjusted *p*-value, is plotted on the y-axis. Yellow and cyan dots indicate genes that have positive and negative interaction values, respectively (right and left quadrants, respectively). The interactions are labeled according to the test item, nicotine concentration (μg/L), sampling time point, gender [F(emale)/M(ale)], and the number of independent samples (mice). For example, “Inter 3R4F Med (13.4 μg/L) 18m F, *n* = 13” represents the interaction term for group of 13 female A/J mice exposed to 3R4F CS at a nicotine concentration of 13.4 μg/L for the 18-month time point.

The interaction model was not fitted separately for every gene, but was fitted by using the popular R package limma which is widely used in gene expression analysis. limma uses moderated t-statistic (t), which is the ratio of the M-value to its standard error. The moderated t-statistic has the same interpretation as an ordinary t-statistic except that the standard errors have been moderated across genes, effectively borrowing information from the ensemble of genes to aid with inference about each individual gene (Ritchie et al., 2015). The number of independent samples (mice), n, is shown together with the contrast name in Figure 1. The raw *p*-values of the interaction coefficient were corrected applying the false discovery rate (fdr) method, and adjusted *p*-values below 0.05 were considered significant. Because there was only 1 spontaneous tumor sample, which did not pass the QC, from month 5, the interaction analysis was restricted to samples from dissection months 10, 15, and 18. The resulting interaction terms were displayed as volcano plots in Figure 1, in which the *x*-axis represents the estimated effect (the interaction coefficient), and the *y*-axis represents the –log_10_(fdr-corrected *p*-value of the interaction coefficient) for each gene.

### Gene Signature Generation

To identify a specific tumor gene signature that discriminates between spontaneous tumors in sham animals and those that were exposure-related, the above statistical model was applied to group MS-300 for data in the previous A/J mouse study (Luettich et al., 2014). Genes were ranked based on the absolute values of the interaction coefficients β_1, *i*_. With only 17 available spontaneous tumor samples in the current A/J mouse study, the maximum number of genes with which a robust covariance could be estimated in a 10-fold cross-validation was 13. The signature is therefore composed of the top 13 genes identified in the previous A/J mouse lung tumor analysis (absolute values of the interaction coefficients >4.8). The size of the gene signature is denoted by *N*. The probability distribution of the spontaneous tumor is described as a multivariate Gaussian distribution, *f* , as follows:


(2) $$f(x_{i})=\frac{1}{\sqrt{{\left(2\pi \right)}^{N}|\Sigma |}}e^{-\frac{1}{2}{\left(x_{i}-u\right)}^{T}\Sigma^{-1}(x_{i}-u)}$$

where *i* is the index of sample i, *x*_*i*_ is the vector of gene expression values, Σ is the covariance matrix, and *u* is the mean of this multivariate Gaussian distribution. The term ${D_{i}^{2}=\left(x_{i}-u\right)}^{T}\Sigma^{-1}\left(x_{i}-u\right)$ in the formula is called the squared Mahalanobis distance (Mahalonobis, 1936). If the covariance matrix is the identity matrix, the Mahalanobis distance reduces to the Euclidean distance. For the purpose of brevity, we refer to the squared Mahalanobis distance simply as the Mahalanobis distance.

The Mahalanobis distance method was used as a 1-class classifier. For any new sample *j*, the squared (“skewed”) distance to the mean (*u*) of the sham group is evaluated by computing the Mahalanobis distance:


(3) $$\begin{array}{r} {D_{j}^{2}=\left(x_{j}-u\right)}^{T}\Sigma^{-1}\left(x_{j}-u\right) \end{array}$$

This distance thereby enables the classification of any sample *x*_*j*_ as a spontaneous tumor if the latter is sufficiently small (please refer to the below classification rule).

The model was trained only on data from the spontaneous tumors of the current A/J mouse lung cancer study. The Mahalanobis distance-based 1-class recall was evaluated by 10-fold cross-validation, iterated 10 times, leading to a 75% recall. This indicates that the distribution is not over-fitted with reasonable confidence. The data from the exposure-related tumors were then used in the model to derive probability estimates for the distance to the sham group tumors. Given the mean and covariance matrix, the squared Mahalanobis distance of all data points follows a χ2 distribution with N degrees of freedom. The classification rule is defined as follow: If the likelihood of a sample Mahalanobis distance according to the above χ^2^ distribution is smaller than 0.05, the tumor was believed to belong to the non-spontaneous group, otherwise, the tumor sample would be classified as spontaneous tumor. The distances were estimated for all samples, and the mean distances were displayed as a bar plot (Supplementary Table 2). We applied the classification rule to 3R4F CS-related lung tumors and tumors from THS 2.2 aerosol-exposed mice.

### Gene Ranking

The human orthologs of the mouse gene signature were obtained using HGNC Comparison of Orthology Predictions (HCOP) (https://www.genenames.org/tools/hcop/). RNA-Seq data for human lung adenocarcinoma samples were obtained from The Cancer Genome Atlas Program (TCGA Research Network; https://www.cancer.gov/tcga). Tumor samples in TCGA data were filtered out if their diagnosis was not “Lung Adenocarcinoma,” if the information from the “tobacco_smoking_history” and “tobacco_smoking_history_indicator” fields were not consistent, or if the content of column “tobacco_smoking_history” was either empty or listed as “Current Reformed Smoker, Duration Not Specified.” We thus retrieved data from 205 tumor samples from 45 current smokers, 130 former smokers, and 30 never-smokers.

The ranks of signature genes in this A/J mouse study and the TCGA human lung adenocarcinoma gene expression data were computed as follows. The interaction terms for the A/J 3R4F group at month 18 were sorted in descending order based on their absolute values. Comparisons of the signs of the signature gene interaction terms in the current A/J mouse study with those in the previous study confirmed that they are 100% consistent. Then, the interaction terms in the TCGA dataset were computed and sorted in descending order based on their absolute values. The signs of the interaction terms of the signature genes in the TCGA dataset were also compared with those in the previous A/J mouse study, and they are 85% consistent. Next, the median ranks of the signature genes in the current A/J mouse study were calculated. To estimate their *p*-values, a bootstrap approach was performed by randomly selecting N genes 10,000 times, and the density of the resulting median ranks was estimated. Similarly, the median ranks of signature genes in the TCGA dataset were estimated, and a density was estimated based on 10,000 times resampling. Additionally, the first quartile (Q1) of the Mahalanobis distance of the 3R4F group/current smoker group minus the third quartile (Q3) of the Mahalanobis distance of the sham group/never-smoker group was estimated for the current A/J mouse and TCGA datasets, respectively. Again 10,000 random re-samplings were performed to obtain the bootstrapped *p*-values.

### Cancer Outlier Gene Analysis

The cancer outlier gene (COG) analysis reported by Seo et al. (2012) was applied to all 17,473 genes on the GeneChip® Mouse Genome 430 2.0 Array across a total of 252 tumor and parenchyma samples. First, all gene expression values were subtracted by their median (location normalization). All expression values were then divided by their 1.4826 × median absolute deviation (scale normalization). Given a set of normalized expression values, Q75 + 3 × inter-quartile range (IQR) is defined as an outlier cutoff, where Q75 is the 75th percentile expression value, and the IQR is the absolute difference between the 25th and the 75th percentile expression values. An expression value was treated as an outlier when its normalized expression value exceeded the outlier cutoff. Finally, genes that exhibited an outlier pattern in at least 1 cancer sample were chosen as candidate COGs.

### DNA Sequencing Analysis

Sample randomization was performed prior to DNA extraction as a complete block randomization, where the blocking factor was defined by both the type of exposure (study group) and the dissection time point.

DNA was isolated from the LCM tissues following the addition of 375 μL AMPure XP magnetic beads (Beckman Coulter Inc., Brea, CA, USA) to each sample and incubation for 15 min on a rotary shaker. The samples were then placed on a magnetic rack for 5 min. Two washes with 1,400 μL 70% ethanol were performed before eluting the captured DNA with 22.5 μL AE buffer (QIAamp DNA Mini Kit, QIAGEN). DNA quantity was assessed on a Qubit® 2.0 fluorimeter (Thermo Fisher Scientific). Two tumor samples failed DNA QC and were therefore excluded from further processing and analysis (Supplementary Table 5).

DNA sequencing libraries were prepared using the Nugen Ovation® Ultralow Library Systems (Tecan Genomics, Inc., Redwood City, CA, USA) following the manufacturer's instructions. The concentrations and sizes of the sequencing libraries were verified on the Agilent 2100 Bioanalyzer. Normalized libraries were pooled in multiplexes of libraries and clustered on Illumina HiSeq 3000/4000 PE flow cells using Illumina HiSeq 3000/4000 PE Cluster Kits (Illumina, San Diego, CA, USA). Sequencing was performed on an Illumina HiSeq 4000 system using Illumina HiSeq 3000/4000 SBS kits (300 cycles).

Reads were cleaned of adapters and trimmed to a maximum length of 150 bases using the bbduk tool version 37.99 (Bushnell, 2014). By using the FastqToSam command from the Genome Analysis Toolkit (GATK) v4.0.1.1s (DePristo et al., 2011), reads were annotated with metadata such as the read group name, flowcell identifier, and lane number as a unique tag. Subsequently, the annotated reads were converted to Fastq format with the GATK SamToFastq tool. The tagged reads were aligned to the mouse genome (m38, Ensembl release 78) by using the BWA MEM algorithm v0.7.17 (Li, 2013), and the mapping was complemented with the GATK MergeBamAlignment tool. The resulting alignment [Binary Sequence Alignment Map, (BAM)] files were filtered for duplicates using the GATK MarkDuplicates tool (DePristo et al., 2011). All individual BAM files for each sample were merged into a single file.

A masking file was created to avoid single nucleotide polymorphism (SNP) calling in areas of very high coverage. For the most densely sequenced female and male parenchyma tissue samples, the sequence coverage density distribution was determined with the SAMtools suite mpileup program. The following procedure was used to generate the mask for excluding high coverage regions: for the autosomes and the X chromosome, the 95th percentile of the read density distribution derived from the female parenchyma sample was chosen as the cut-off; for the Y-chromosome, the 95th percentile of the read distribution from the male parenchyma sample was chosen. Sites in the genome with more than the specified coverage were identified, and contiguous sites were joined to segments. These segments were filtered by a minimum length of 20 bp, extended on either side by 50 bp, before merging adjacent elements closer than 30 bp. The resulting exclusion list was inverted using the bedtools complement function (Quinlan and Hall, 2010) to yield a “whitelist.”

The merged BAM files were grouped by animal, whereby at least 1 tumor sample and 1 parenchyma sample had to be present in the group for the calling to be performed. These groups were used as input for Freebayes v1.2.0 (Garrison and Marth, 2012), which called variants based on the whitelist. This resulted in 1 file (Variant Calling Format [VCF]) per group containing the joint calls for all input BAM files. Initially, heterozygous SNPs were selected from the VCF file if they had a minimum quality score of QUAL>1. Subsequently, mutations were selected that were specific to the tumor tissue (i.e., they occurred in the tumor tissue, and there were no reads supporting the presence of this mutation in the parenchyma tissue). If any of the parenchyma samples had even a single read supporting the call, the mutation was deemed pre-existing. The mutations were annotated using CAVA v1.2.3 (Munz et al., 2015) to determine the genic effect of the mutation. For point mutation analysis, heterozygous mutations with a CIGAR string equal to 1X and an ODDS score > 10 were selected. Allosomal mutations were excluded from the mutation spectrum analysis to assure comparability between the samples. For the functional analysis, all heterozygous mutations were used, except for the allosomes in males, in which case only homozygous mutations were selected.

Mutations were processed in R (v3.2.2 for data processing, v3.4.3 for visualization) using the VariantAnnotation (Obenchain et al., 2014) and SomaticSignatures (Gehring et al., 2015) packages. Mutation spectra were calculated by counting each of the mutation types [following their conversion to the pyrimidine first notation (C → A, C → G, C → T, T → A, T → C, T → G)] and converting them to percentages of the total per sample. Analogous to the gene expression data, the Mahalanobis distance of each sample to the centroid of the sham group was calculated, but using the vector of mutation type frequency instead of the vector of gene expression values; 1 column (arbitrarily, the T > G mutation column was chosen) was excluded from the analysis to avoid collinearity amongst the input variables (Supplementary Table 3).

For clustering, distances between the samples based on the mutation spectra were calculated using the dist function in R (default Euclidian distance), and the distance matrix was used as the input for hierarchical clustering, as implemented in the R function hclust (the default complete linkage method was used).

### Statistical Analysis

The comparisons between the means of Mahalanobis distances in different groups for both gene expression data and mutational spectra were performed by 2-sample Student's *t*-test with Welch modification to the degrees of freedom. Specifically, the R function *t*-test was used (Ripley, 2001). *P* < 0.05 were considered significant.

## Results

### Gene Expression

To delineate the differences between lung tumors forming spontaneously in air-exposed A/J mice and tumors present in animals following exposure to 3R4F CS or THS 2.2 aerosol, we employed an interaction analysis, taking into account the 2 tissue types, tumor and parenchyma, and the exposure effects relative to air exposure. This highlighted 7 significantly differentially expressed genes (based on interaction terms) in the lung tumors of animals exposed to 3R4F CS for 18 months compared to sham animals (fdr-adjusted *p* < 0.05). Of these genes, 1 (*Arsb*) was upregulated and 6 (*Lcn2, Cxcl1, Rgs1, Lrg1, Lhfpl2, Msr1*) were downregulated in tumors from CS-exposed animals compared to spontaneous tumors from sham animals (Figure 1).

The interaction analysis did not identify any differentially expressed genes between the lung tumors in THS 2.2 aerosol-exposed and sham-exposed mice (Figure 1).

Our previous analysis of A/J mouse lung tumor gene expression profiles indicated a suppression of the humoral immune response in tumors from 3R4F CS-exposed animals, with an overall decrease in expression levels of genes contributing to the humoral immune response network and a predicted reduction in B cell function (Luettich et al., 2014). At the same time, gene enrichment analysis suggested enhanced accumulation of glycosphingolipids, glycosylceramide, glycosaminoglycans, and lipids in CS exposure-related tumors compared to spontaneous tumors, while processes contributing to cellular homeostasis of lipid metabolites, such as transport, efflux, and degradation, as well as the expression of multiple lysosomal enzyme-encoding genes, appeared to be suppressed following exposure. We suspected an intricate interlinking of these processes resulting in perturbations of the anti-tumor immune response, with insufficient antigen presentation potentiating the ability of tumor cells to escape from immune surveillance in CS-exposed A/J mice (Luettich et al., 2014). In the current study, gene expression profiles of lung tumors from CS-exposed mice exhibited similar features, including suppressed immune response, decreased leukocyte activation, migration, adhesion and infiltration (z-scores: −2.997 to −1.99), and increased lung inflammation (z-score: 1.311) (Supplementary Table 4). However, an obvious decline in B cell function based on gene expression analysis was not apparent, although a number of genes implicated in B cell proliferation (e.g., *Ccl28, Ccr6, Cd44, Cd80, Cd86, Ctsb, Fcgr1b, Fcgr2b*, etc.) were downregulated in tumors from CS-exposed mice compared to those from sham-exposed mice. Similarly, marked effects on lipid or glycophospholipid metabolism pathways were not observed. The absence of statistically significant changes in gene expression levels in lung tumors from THS 2.2 aerosol-exposed mice precluded a similar analysis of affected pathways and biological processes.

### Tumor Classification

**Figure 2:**
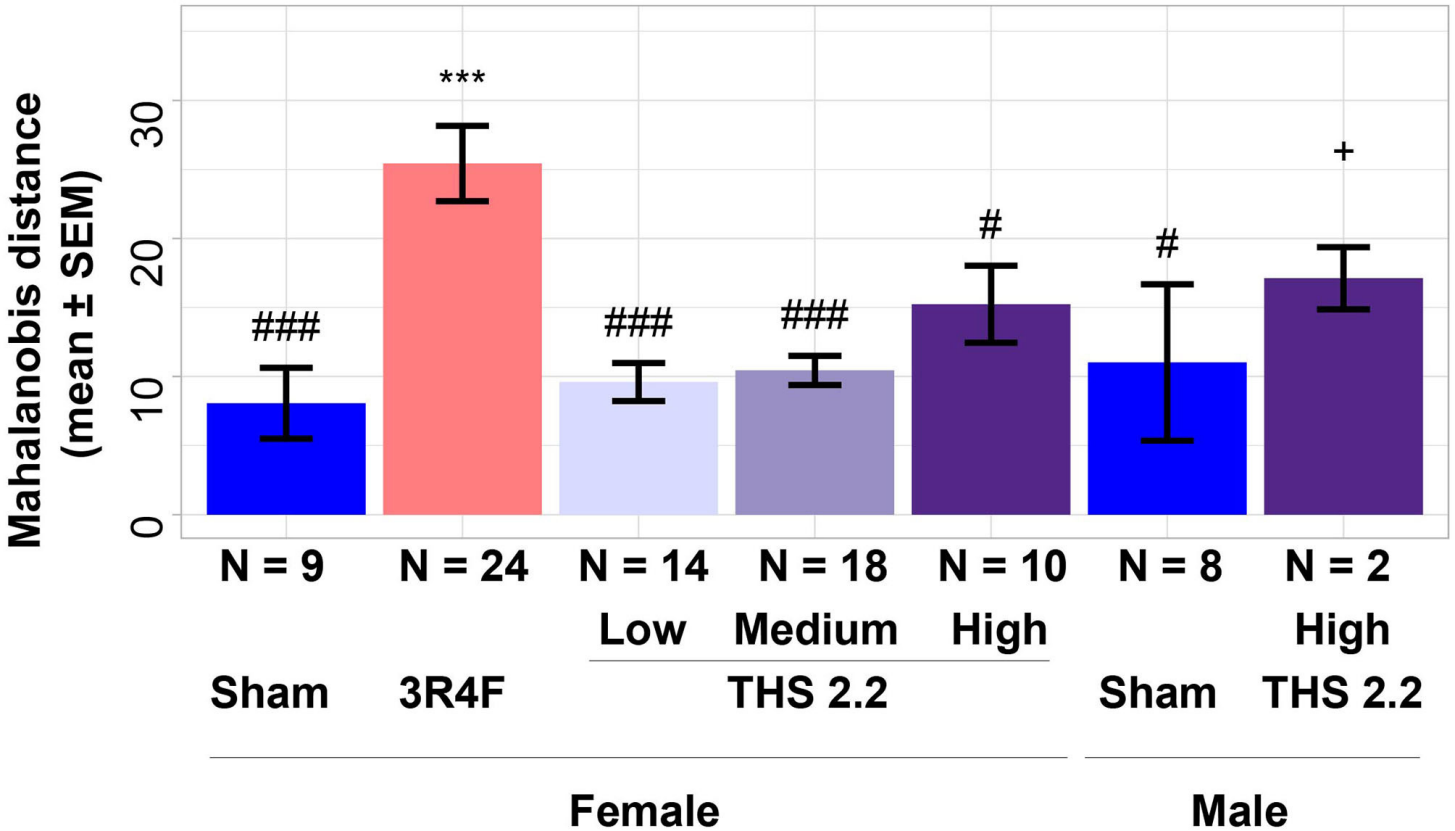
Estimates of similarity between lung tumors in A/J mice using gene signature data. As an estimate of similarity between lung tumors, the Mahalanobis distance between lung tumors in sham animals and those in each exposure condition (indicated on the x-axis) was calculated based on a 13-gene signature derived from the interaction analysis of gene expression data from a previous A/J mouse lung cancer study (E-MTAB-1871). Results are presented as mean ± standard error of the mean (SEM). Significant differences between exposure and sham groups are represented by ***(*p* < 0.001); significant differences between THS 2.2 aerosol and 3R4F CS exposure groups are represented by #(*p* < 0.05) and ###(*p* < 0.001). + Indicates that there were only 2 tumor samples in this study group. THS, Tobacco Heating System. The number of QC-passed tumor CEL files, N, for different groups are given under the corresponding bars.

To further investigate differences between lung tumors arising in sham-exposed mice compared to those in 3R4F CS- or THS 2.2 aerosol-exposed A/J mice, a 1-class classifier was derived from gene expression data of the previous A/J mouse lung cancer study (Luettich et al., 2014). This classifier comprised the highest ranked genes (absolute interaction value > 4.8): *Scgb3a1, Iglv1, Ighv1-14, Bex1, Ighg3, Chia1, Ighm, Ighg2b, Iglc1, Saa3, Acoxl, Itih*4, and *Ighg1*. These 13 genes were not indicative of an exposure effect, because the interaction analysis accounts for exposure effects. This gene signature was then used to calculate distances and the associated probability estimates of similarity between tumors in 3R4F CS- or THS 2.2 aerosol-exposed and sham animals. Based on the 13-gene signature, the results showed that lung tumors in 3R4F CS-exposed mice were significantly different from those in air-exposed animals (*p* < 0.001). In addition, based on this gene signature, lung tumors from female THS 2.2 aerosol-exposed mice were not significantly different from those in sham animals. They were, however, significantly different from those in 3R4F CS-exposed mice [*p* < 0.001 for THS 2.2 L (6.7 μg nicotine/L) and THS 2.2 M (13.4 μg nicotine/L); *p* < 0.05 for THS 2.2 H (26.8 μg nicotine/L)]. The lung tumors from male A/J mice exposed to THS 2.2 aerosol also appeared to exhibit dissimilarities to tumors in sham and 3R4F CS-exposed mice. However, because the number of tumors in the male THS 2.2 aerosol-exposed mice was small (*N* = 2), the statistical test was not as powerful as that for the corresponding female study group (Figure 2).

**Figure 3:**
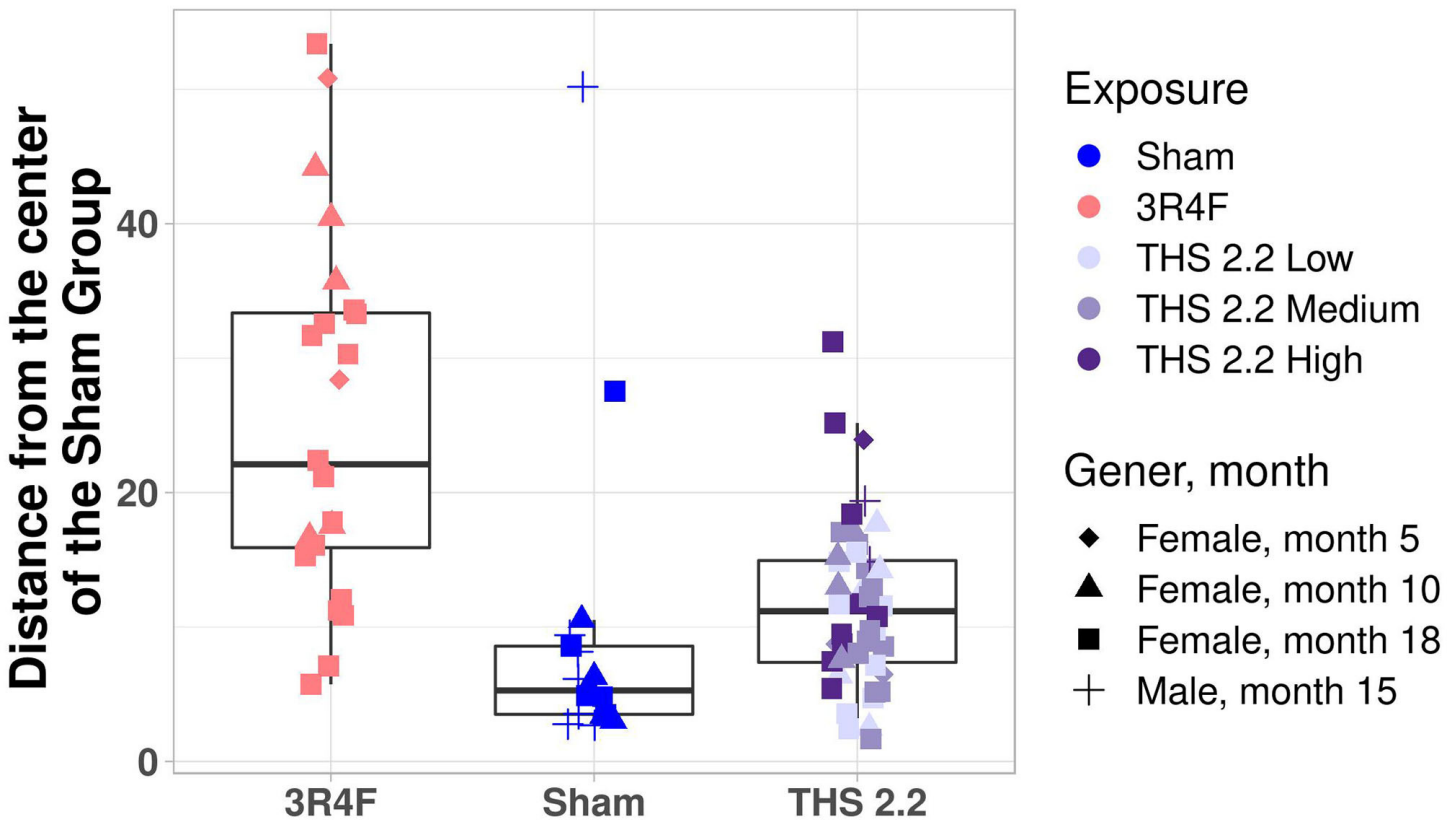
Mouse lung tumor classification. Mahalanobis distance of each individual tumor in 3R4F CS- or THS 2.2 aerosol-exposed mice from the center of the sham group using the 1-class model based on the gene expression data. Exposure groups are indicated by color; sex and time point are indicated by different symbols (see legend). The Mahalanobis distances between the gene signature for each tumor sample and those of the sham group are presented as box plots. The centerline represents the median, and the box encloses the 1st and 3rd quartiles (“hinges”). The upper and lower whiskers represent the furthermost points from the respective hinges, which are no more than 1.5 IQRs from the hinge. Data are shown as individual points. THS, Tobacco Heating System.

To better visualize the differences between the 2 classes of tumors on a tumor-by-tumor basis, the similarity measure was also visualized as a box plot (Figure 3). This data view clearly indicates that, based on the gene signature, the majority of lung tumors in THS 2.2 aerosol-exposed mice were similar to the lung tumors in sham animals but different from those in 3R4F CS-exposed mice. In addition, 2 extreme values became apparent among lung tumors from the sham group, which were collected from 1 male and 1 female mouse at month 15 and month 18, respectively (Figure 3).

### Gene Signature Translatability

Because the gene signature was developed from mouse lung tumor data, and the A/J mouse is a model of CS-related lung cancer in humans, the question of translatability and applicability of the signature to human lung tumors arose. Therefore, we examined the ability of the gene signature, once orthologized, to discriminate lung adenocarcinomas in smokers from those in never-smokers using gene expression data from TCGA (Cancer Genome Atlas Research Network, 2014).

**Figure 4:**
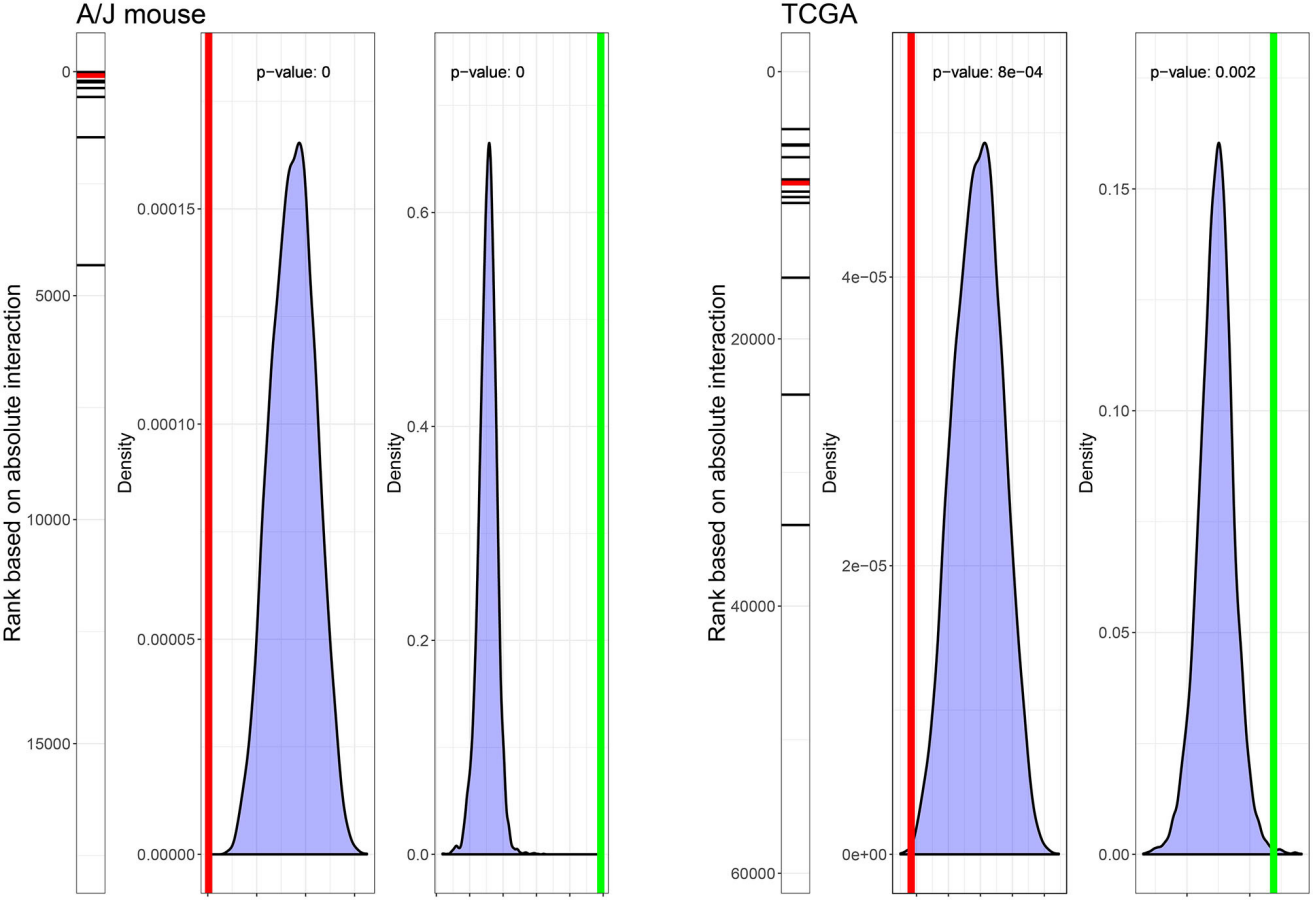
Ranks and statistical characteristics of the gene signature in mouse and human lung tumor gene expression data. The rank of the 13 signature genes, the density plot of 10,000 median ranks of 13 randomly selected genes, and the density plot of ΔQ1–Q3 (3R4F/smoker, sham/never-smoker) based on 10,000 sampling of 13 random genes in the dataset of the current A/J mouse lung cancer study (left panel) and the TCGA dataset (right panel) are displayed. Red lines indicate median ranks of gene signature genes; green lines indicate ΔQ1–Q3 (3R4F/smoker, sham/never-smoker).

First, we evaluated the enrichment of the signature genes in the interaction term values from the current A/J mouse study and the TCGA dataset. The gene signature ranked high in both the current A/J study and human TCGA datasets, with *p*-values of the median rank of 0 and 0.0008, respectively. Then, we evaluated the specificity of the signature with respect to random sets of 13 genes. *P*-values for ΔQ1–Q3 (3R4F/smoker, sham/never-smoker) were 0 and 0.002 in the current A/J mouse and TCGA datasets, respectively (Figure 4).

**Figure 5:**
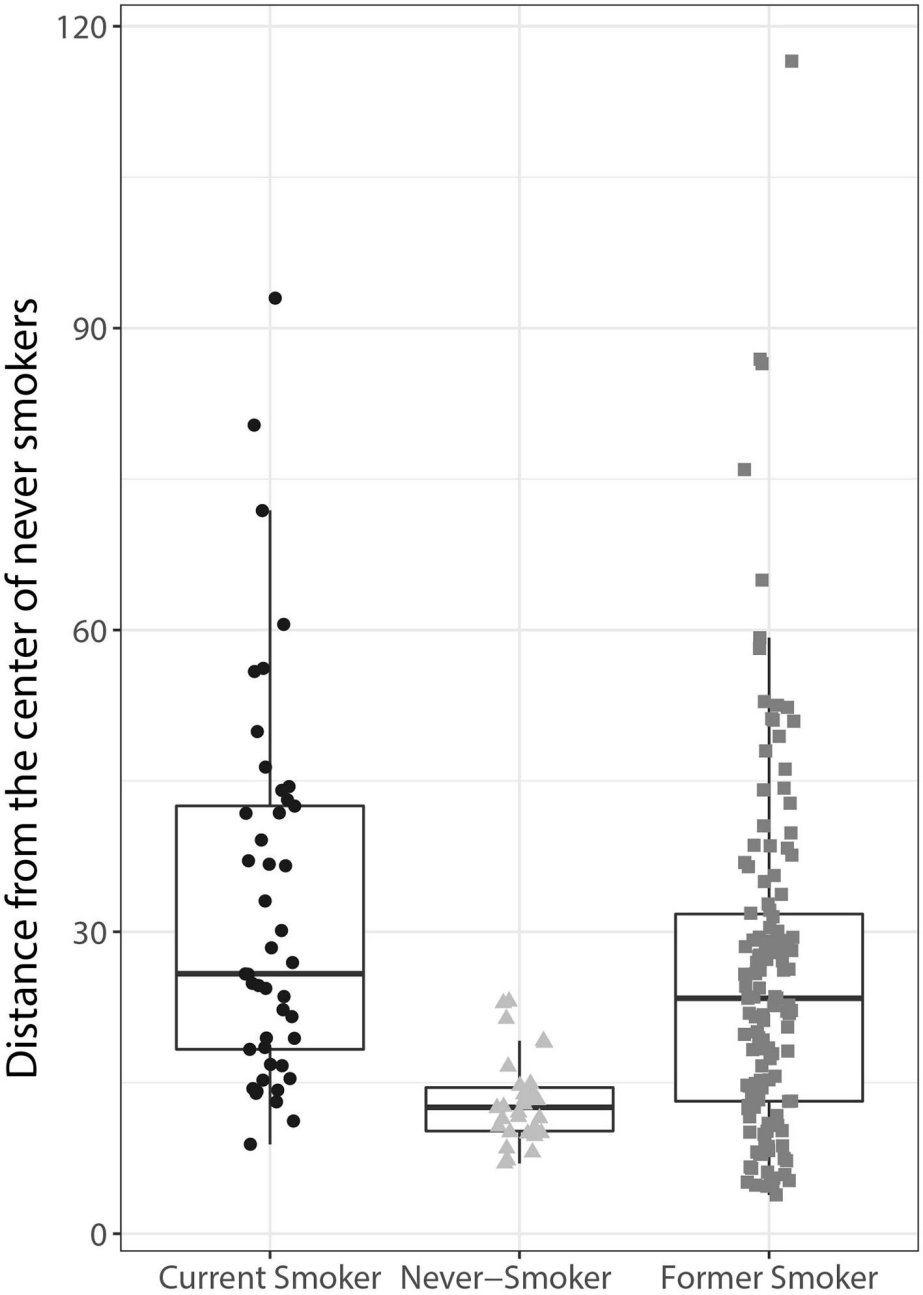
Human lung adenocarcinoma classification using the orthologs of the mouse lung tumor gene signature. The Mahalanobis distances between the gene signatures for each tumor sample are presented as individual data points in box plots for each of the groups indicated on the x-axis (current, former, and never-smoker). The centerline represents the median, and the box encloses the 1st and 3rd quartiles (“hinges”). The upper and lower whiskers represent the furthermost points from the respective hinges, which are no more than 1.5 IQRs from the hinge. Data are shown as individual points.

Applying the orthologized gene signature, the current smoker group separated well from the never-smoker group, and the difference between these 2 groups was statistically significant (*p* < 0.05, *t*-test). Former smokers exhibited similarities to both never- and current smokers with respect to the gene signature, with the median distance closer to the current smokers than to the never-smokers (Figure 5).

Together, these data show that the gene signature derived from the mouse lung tumor data is able to distinguish lung adenocarcinomas in current smokers from those in never-smokers. Of note, the distinction also appears to be technically robust, considering that the gene signature was derived from a microarray gene expression dataset and applied to an RNA-Seq dataset.

### Mutation Spectra

To identify mutations occurring in the mouse lung tumor samples, the sequencing reads from all parenchyma-tumor pairs were mapped to the mouse reference genome. Mutations unique to the tumor samples (i.e., those not occurring in the matched parenchyma tissue) were selected for downstream analysis of the total number, the frequency of base substitution, and their potential phenotypic effects.

**Figure 6:**
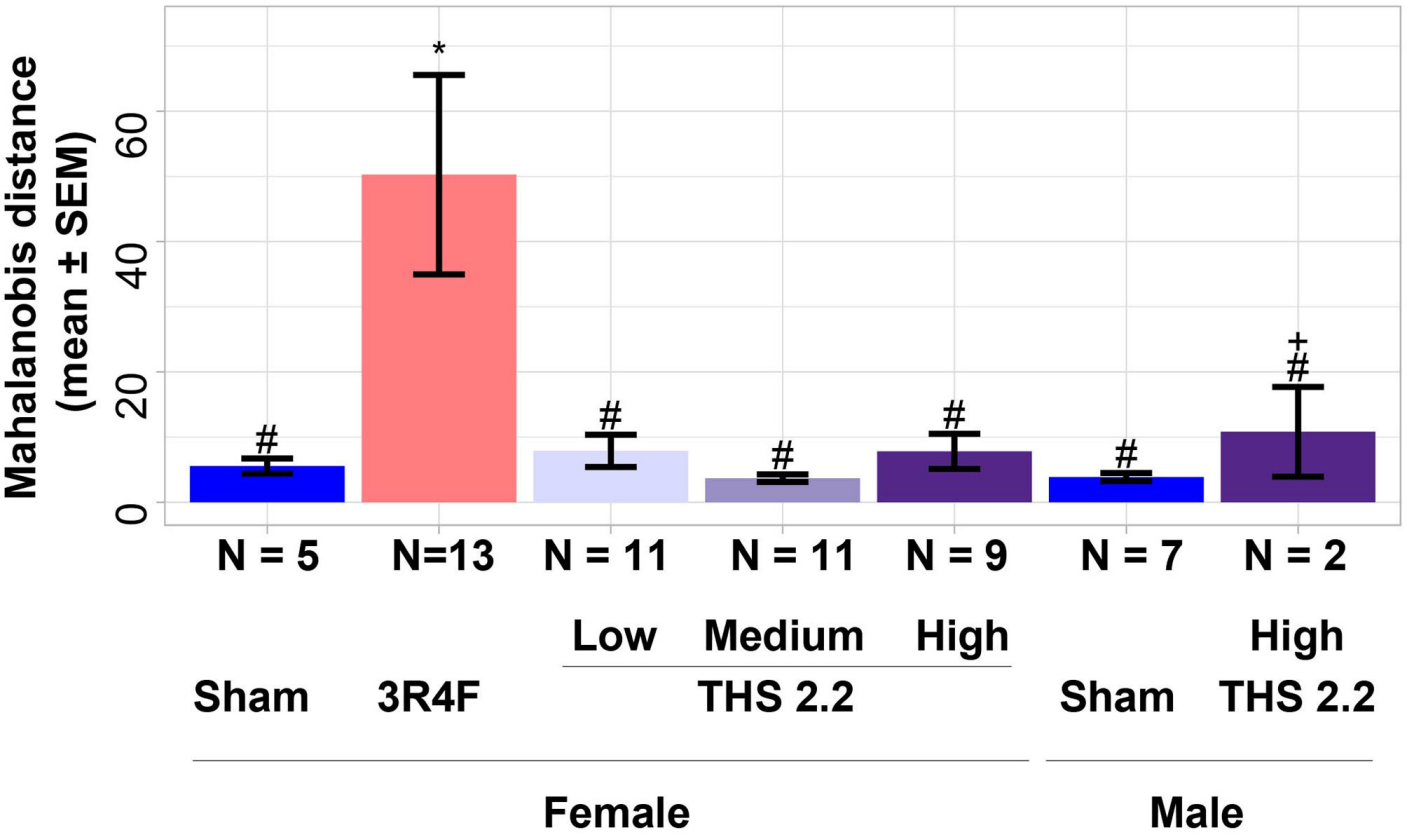
Estimates of similarity between lung tumors in A/J mice using mutation spectra data. As an estimate of similarity between lung tumors, the Mahalanobis distance between lung tumors in sham animals and those in each exposure condition (indicated on the x-axis) was calculated based on mutation spectra. Results are presented as mean ± SEM. Significant differences between exposure and sham groups are represented by *(*p* < 0.05); significant differences between THS 2.2 aerosol and 3R4F CS exposure groups are represented by #(*p* < 0.05). + indicates that there were only 2 tumor samples in this study group. THS, Tobacco Heating System. The number of analyzed samples that could be combined with the transcriptomics samples, N, for different groups are given under the corresponding bars.

Mutation counts per sample were below 2000 for all sham and THS 2.2 and most 3R4F tumor samples. There were, however, 2 samples from the 3R4F treatment group with point mutation counts of 5,026 and 6,626 (Supplementary Figure 2). Next, for each tumor, the frequencies of the 6 types of single-point mutations (C → A, C → G, C → T, T → A, T → C, T → G) were calculated, yielding a mutation spectrum. Other than a small subgroup of 3R4F tumors with a higher proportion of C → A mutations, there was no clear, systematic formation of clusters, in that the tumors from animals of the various treatment groups did not segregate clearly based on mutation spectrum (Supplementary Figure 3). The mutational profile observed at the trinucleotide level in a subset of tumors corresponds with signatures typically associated with, amongst others, lung cancer (Alexandrov, 2015; Supplementary Figure 4). The per-tumor mutation spectrum was therefore used for calculating the Mahalanobis distances between tumors in exposed and sham animals. This analysis showed that the only exposure group that had a significantly different (*p* < 0.05) as well as an increased average Mahalanobis distance to the sham group was the 3R4F CS exposure group (Figure 6). By contrast, the mutational spectra of the tumor samples from THS 2.2 aerosol-exposed mice were not statistically significantly different from those of the sham group (Figure 6).

**Figure 7:**
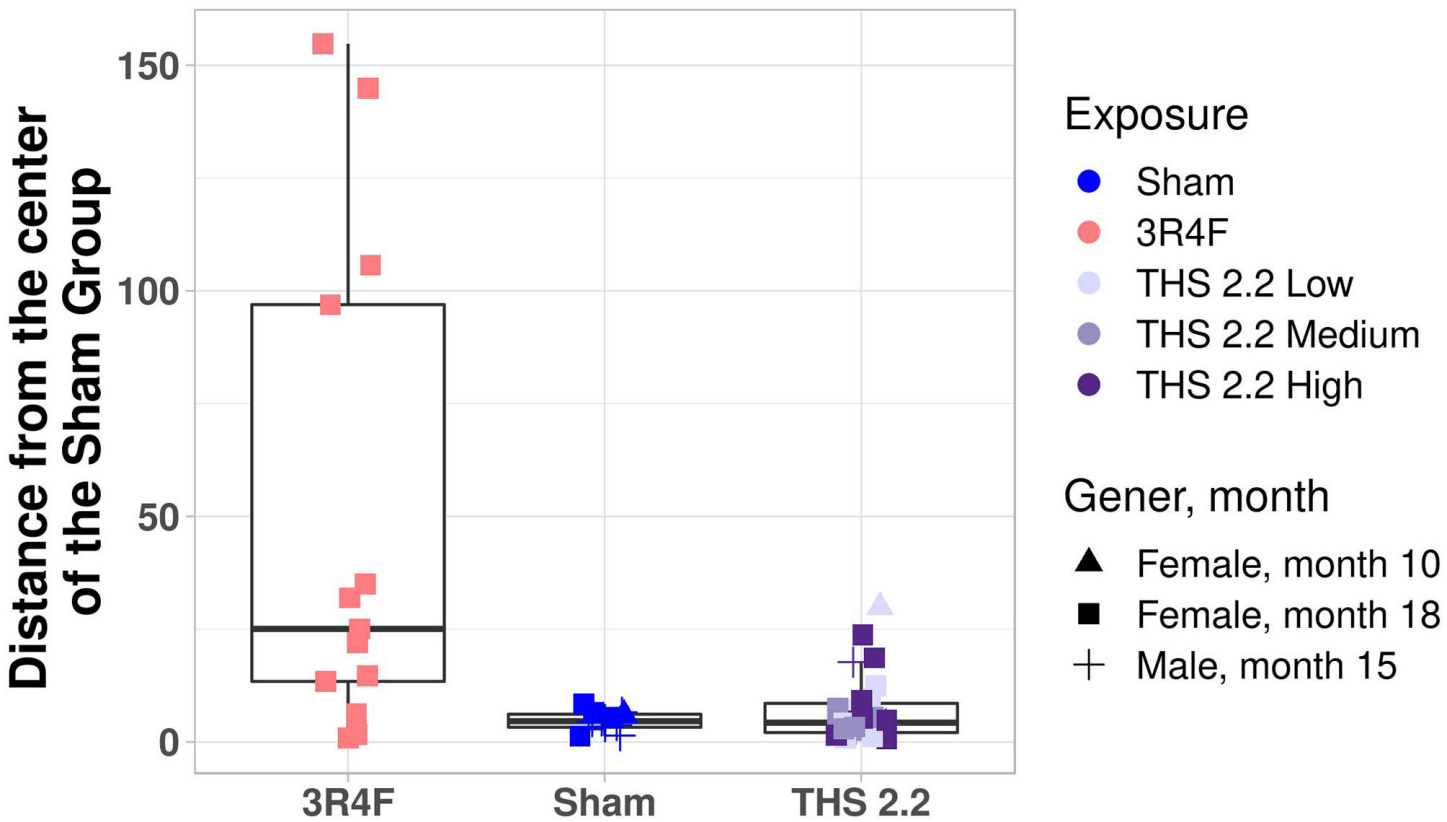
Mouse lung tumor classification. Mahalanobis distance of each individual tumor in 3R4F CS- or THS 2.2 aerosol-exposed mice from the center of the sham group using the 1-class model based on the mutation spectra. Exposure groups are indicated by color; sex and time point are indicated by different symbols (see legend). The Mahalanobis distances between the mutational spectra for each tumor sample and those of the sham group are presented as box-whisker plots. The centerline represents the median, and the box encloses the 1st and 3rd quartiles (“hinges”). The upper and lower whiskers represent the furthermost points from the respective hinges, which are no more than 1.5 IQRs from the hinge. Data are shown as individual points. THS, Tobacco Heating System.

To better visualize the differences between the 2 classes of tumors on a tumor-by-tumor basis, the similarity measure was also visualized as a box-whisker plot (Figure 7). This data view shows that, based on the mutation spectra data, the majority of lung tumors in THS 2.2 aerosol-exposed mice were more similar to the lung tumors in sham animals than to those from 3R4F CS-exposed mice. A robust equivalence test based on the mutation spectra data was performed to test if the lung tumors in THS 2.2 aerosol-exposed mice is significantly similar to the lung tumors in sham animals, compared to the lung tumors from 3R4F CS-exposed mice. The R function rtost in R package equivalence from CRAN was used (Robinson, 2016). The magnitude of region of similarity, epsilon, is chosen to be 5% of the distance between the means of the lung tumors from 3R4F CS-exposed mice and the lung tumors in sham animals. The *p*-value is 0.02, which means that the lung tumors in THS 2.2 aerosol-exposed mice is significantly similar to the lung tumors in sham animals, compared to the lung tumors from 3R4F CS-exposed mice.

Subsequently, the location of the mutations was determined relative to the genes, and a list of genes containing at least 1 mutation was generated. As the total number of exonic point mutations was low, any mutation location (including intronic mutations) was considered to affect genes. Surprisingly, *Kras* point mutations were observed in only 6 lung tumors (3 sham, 1 3R4F, 1 THS 2.2 L, and 1 THS 2.2 H tumor sample), suggesting that point mutation is not the predominant cause for the proposed oncogene activation in 3R4F CS- or THS 2.2 aerosol-exposed mice. *Lrp1b* was the gene most frequently affected by point mutations, followed by *Csmd1, Fgfr2, Grm7, Dcc, Fhit*, and *Csmd3*. There was no obvious relationship between mutation frequency and type of exposure.

**Figure 8:**
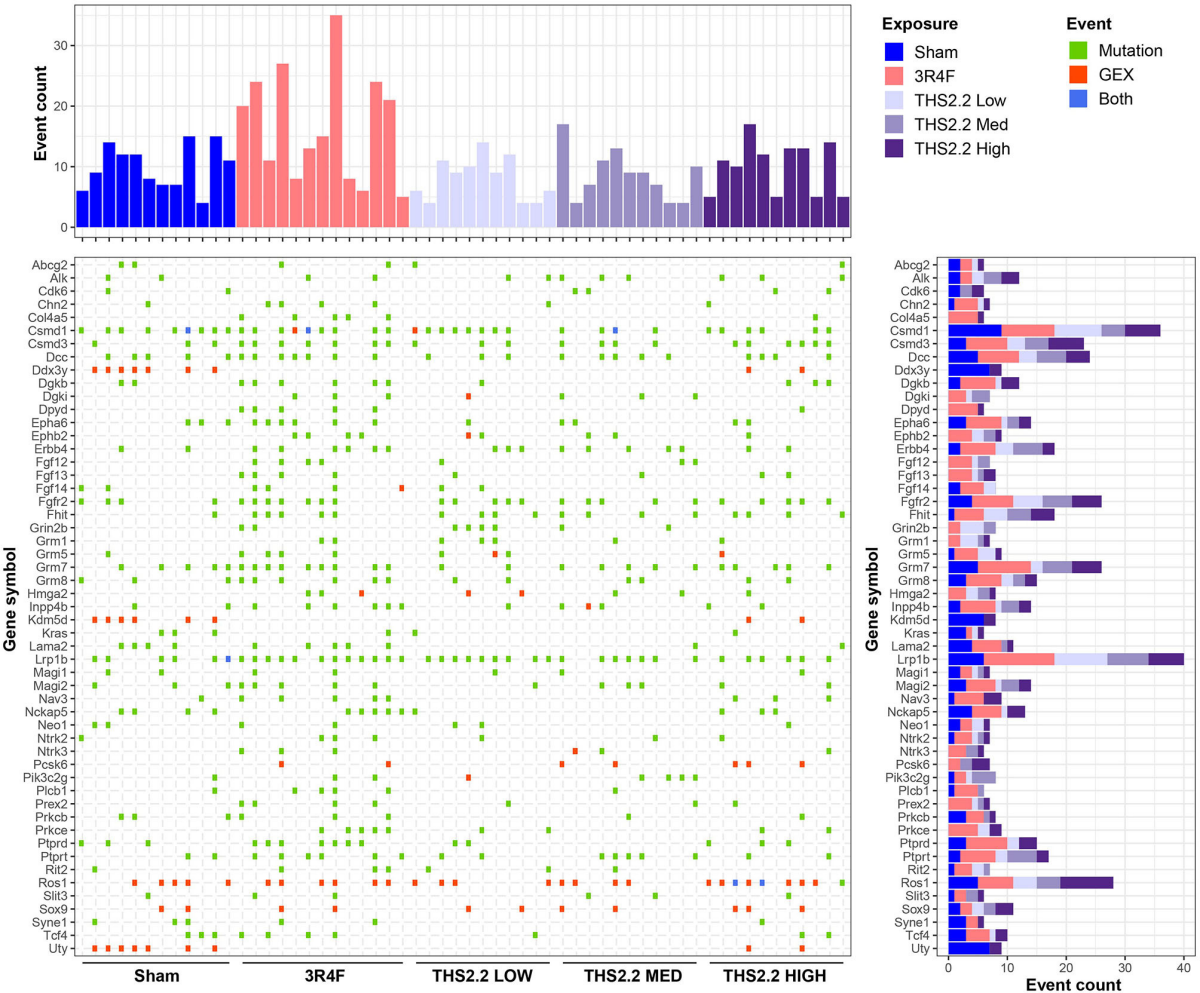
The transcriptional and mutation landscape of A/J mouse lung tumors. An overview of genes affected by mutation (green) or upregulation (red) or both (blue) is presented in a tile plot. The top panel displays the number of affected genes per tumor sample, whereas the panel on the right illustrates the number of affected samples for a given gene; the height and color scheme of the bars indicate the number of events observed per exposure type. GEX, gene expression.

Overall, very few genes had protein function-altering point mutations in more than 1 of the tumor tissues sequenced here, preventing further conclusions to the potential phenotypic effects of these mutations. Therefore, the list of genes affected by mutations was combined with the list of genes considered to be gene expression outliers (COGs), and genes were filtered for their previously reported role(s) in human cancers. This yielded a transcriptional and mutation landscape, providing a unique insight into the molecular makeup of age- and exposure-related tumors in this mouse strain (Figure 8). Most noticeable in this landscape view is that most genes exhibited either a mutation or extreme upregulation, but very rarely both. In addition, *Ros1* expression was frequently highly upregulated, independent of exposure, whereas *Ddx3y, Kdm5d*, and *Uty* gene expression was strongly increased in the same tumors from sham and THS 2.2 High aerosol-exposed mice.

## Discussion

The A/J mouse strain is susceptible to lung tumorigenesis following chemical exposure, including exposure to CS (Coggins, 2010). As this mouse strain consistently shows a significant and cigarette smoke total particulate matter concentration-dependent increase in lung tumor incidence and multiplicity after 15 to 18 months of exposure (Stinn et al., 2013a,b), it has been historically used in lung tumorigenesis studies. This body of work helped us understand the temporal pattern of emphysema and tumor development, as well as tumor progression in A/J mice, and led us to consider the A/J mouse as an appropriate model for lung tumorigenesis (Wong et al., 2020). Although CS exposure leads to increased lung tumor incidence and multiplicity, non-exposed A/J mice will also develop lung tumors spontaneously as they age (Witschi, 2004). The majority of murine lung lesions are classified as hyperplasias and adenomas, which lack the histological heterogeneity that is seen in human lung carcinomas (Nikitin et al., 2004). Moreover, histologically, spontaneous tumors are indistinguishable from chemical exposure-related tumors. This raises the question whether the exposure-related tumors are of a similar type to spontaneous tumors and whether molecular characterization of these 2 tumor types could provide additional insights in support of human hazard estimations. The distinction becomes particularly important when the overall tumor response is moderate and the dose-response is shallow, as is the case in the A/J mouse model (Stinn et al., 2013a). Our previous study indicated that gene and miRNA expression analyses of A/J mouse lung tumor tissues may be useful means to delineate potential mechanisms that underpin the divergence of tumor progression in CS-exposed mice from that in air-exposed mice (Luettich et al., 2014).

Given our prior experience with this lung cancer model, we utilized gene expression profiles of LCM lung tissues to develop a classification approach. Unlike previously, we argued that as exposed mice may exhibit both exposure-related and spontaneous tumors, distinguishing the 2 should be considered a 1-class rather than a 2-class problem, with the only unequivocally defined class being the spontaneous tumors arising in sham animals. One-class classification, which is also known as unary classification or class-modeling, aims at identifying samples of a specific group amongst all samples by learning from a training set containing only the objects of that group (Désir et al., 2013; Irigoien et al., 2014; Oliveri, 2017). Because in many cases groups are not ambiguous, there are far fewer applications of 1-class classification than of 2- or multi-class classification in biomedical studies (Yang et al., 2012; Ganesan et al., 2013). Applying an interaction analysis to gene expression data, we extracted a gene signature that can be used as a tumor classifier surmising that, as only spontaneous tumors arising in sham-exposed animals are unequivocally defined, a 1-class classification can be applied. The methods for 1-class classification can be divided into 3 groups: density estimation, boundary methods, and reconstruction methods. The Mahalanobis distance method is a density estimation, which is simpler and more robust for data sets with different covariance structure and a more natural choice for gene expression data. Bias in gene signatures (i.e., lower true classification accuracy than the reported classification accuracy) is a common challenge in gene signature generation. A review of 111 high-impact manuscripts involving classification analysis of gene expression data found that 58 (53%) drew their conclusions based on a statistically invalid method, which can lead to bias in a statistical sense (Barbash and Soreq, 2013). In our approach, gene signature bias was addressed by leveraging gene expression data from an independent A/J mouse study to develop a tumor gene signature (Luettich et al., 2014). Of note, the gene signature and the 1-class classifier successfully discriminated spontaneous tumors in sham animals from exposure-related tumors in this A/J mouse lung cancer study with 75% recall in a 10-times 10-fold cross-validation. In addition, the classification also indicated that tumors from THS 2.2 aerosol-exposed mice were not significantly different from those of sham animals, suggesting a lack of carcinogen-driven divergence in those tumors, which could be a direct consequence of the significant reductions in carcinogen levels in THS 2.2 aerosol. To examine the translatability of these findings, the gene signature (orthologized based on gene symbol) was then tested on human lung adenocarcinoma gene expression data from the TCGA database (https://www.cancer.gov/tcga). This analysis showed that lung tumors in never-smokers could be discriminated from lung tumors in former and current smokers, indicating that both gene signature and classifier are robust and translatable from mice to humans.

There are major clinical differences between lung cancers arising in never-smokers and smokers and their response to targeted therapies. Non-smoking status is actually the strongest clinical predictor of benefit from EGFR tyrosine kinase inhibitors (Sun et al., 2007). Even though our signature discriminated cancers in never-smokers from the majority of cancers in ever-smokers, there were some tumors in ever-smokers which are similar in gene expression profile to tumors in never-smokers, as demonstrated by the Mahalanobis distances (Figure 5). There is a possibility that these patients, even though they are ever-smokers, may also benefit from treatment with EGFR tyrosine kinase inhibitors. This 13-gene gene signature could enable the development of a cost efficient PCR kit for identifying these patients.

In addition to gene expression profiling, we also explored DNA mutation profiles of spontaneous and exposure-related lung tumors in this mouse model. Mutation data were used for classification tasks in the past (Alexandrov et al., 2013; Alexandrov et al., 2016b; Phillips, 2018). With recent advances in cancer genome sequencing characteristic mutation signatures can be derived from different cancer types. For example, sequencing of a small cell lung cancer cell line yielded characteristic CS exposure-related mutation patterns (Pleasance et al., 2010). A subsequent large-scale sequencing effort showed that these exposure effects were indeed consistent across multiple cancer genomes, leading to the identification of a CS-specific mutational signature (Alexandrov et al., 2016a), which is thought to recapitulate the processes involved in mutagenesis (Nik-Zainal et al., 2015). Therefore, we extended tumor classification to include mutation spectra using a similar 1-class approach to the one applied to gene expression data. This approach showed that mutation spectra were also different between lung tumors from 3R4F CS-exposed mice and those in sham animals, and again, tumors from THS 2.2 aerosol-exposed mice resembled those from air-exposed mice. This is an important finding considering that the number of A/J lung tumor mutations was relatively small compared to the high frequency of mutations seen in human non-small cell lung cancers (Cancer Genome Atlas Research Network., 2012, Cancer Genome Atlas Research Network, 2014). Moreover, the current findings also indicated that K-ras activation, postulated to predispose A/J mice to lung tumor formation (Lin et al., 1998), may not occur via point mutation. This contrasts with human lung adenocarcinomas, in which mutant K-ras is thought of as oncogenic driver and of which between 19 and 33% were shown to harbor oncogenic *KRAS* mutations (Cancer Genome Atlas Research Network, 2014; Wu et al., 2015). It is possible that the small sample size in this study comprising all proliferative lesions rather than only lung tumors masked a potential effect on *Kras*.

Based on the molecular tumor landscaping attempted here, it is tempting to speculate that other known cancer genes are involved in driving lung tumorigenesis in this mouse strain. For example, *Lrp1b*, a putative tumor suppressor, was identified as the gene most frequently affected by point mutations, without an obvious link to the type of exposure. *LRP1B* mutations were also described in atypical adenomatous hyperplasias, which are precursors of human lung adenocarcinomas (Park et al., 2018). Moreover, *LRP1B* mutations were found to be overrepresented in lung adenocarcinomas in chronic obstructive pulmonary disease patients, independent of smoking status (Xiao et al., 2017). Similarly, *FGFR2* mutations residing in the gene's kinase domain are a frequent observation in human non-small cell lung cancers, even in the absence of prior CS exposure, and led to lung adenocarcinoma formation in conditional knock-in mice (Tchaicha et al., 2014). Together, these findings suggest that *Lrp1b* and/or *Fgfr2* may be linked to the propensity of tumor formation in the A/J mouse model of lung cancer. Other genes, such as *Dcc, Csmd1, Csmd3*, and *Fhit*, were also frequently affected in lung tumors from sham and CS- or THS 2.2 aerosol-exposed mice. While mutations in *Dcc, Csmd3*, and *Fhit* are rather uncommon in human lung adenocarcinomas, deletions or allelic imbalances occur frequently and are considered early events in human lung tumorigenesis (Sozzi et al., 1998; Kohno et al., 2000; Ma et al., 2009; Ahn et al., 2014; Cancer Genome Atlas Research Network, 2014). Little is known about the role of the *CSMD* genes in the context of lung cancers. Previous studies suggested, however, that neither *Fhit* nor *Dcc* genetic alterations confer increased susceptibility to lung tumor formation in mice (Fazeli et al., 1997; De Flora et al., 2007), pointing to a potentially novel mechanism that may be specific to the mouse strain used in this study. Finally, *Ros1* overexpression was more frequently seen in lung tumors of A/J mice than mutations. *ROS1* gene rearrangements occur in 1–2% of human non-small cell lung cancers and confer a distinct clinical phenotype (Bergethon et al., 2012; Gainor and Shaw, 2013). While *ROS1* expression was reportedly increased in absence of translocation events (Lee et al., 2013), fusions of *ROS1* with other genes may also give rise to increased transcript levels (Li et al., 2011; Kalla et al., 2016). It is therefore possible that rearrangements involving *Ros1* occurred in our sample set of murine lung tumors. Additional tests (e.g., with fusion-specific probes) will be necessary to further elucidate this observation.

It is worth noting here that this study has some limitations. The inhalation study design was aligned with the OECD test guideline 453 (OECD, 2018) to meet the minimum animal numbers required for cancer endpoints at terminal dissection (*N* = 50 per group). However, it was not possible (for both ethical and technical/logistical reasons) to include additional animal groups of that size to accommodate omics investigations. Since lung tumor incidence and multiplicity in THS 2.2 aerosol-exposed animals were not different from those in sham animals (Wong et al., 2020), the resulting sample set was small. Therefore, samples were not further divided into groups comprising nodular hyperplasia and true lung tumors (i.e., adenomas and adenocarcinomas), but rather were summarily examined as “lung tumors.” With adequate numbers of biological replicates, sample stratification based on histology may result in an even more comprehensive analysis of A/J mouse lung tumors, with the potential to substantiate progression from hyperplasia to adenoma to adenocarcinoma. In addition, we realize that applying an interaction term to the gene expression data eliminated many of the exposure effects that are typically described in CS studies. In consequence, subsequent enrichment analyses that may give rise to mechanistic interpretations were not possible. Similarly, the number of putative protein-altering point mutations was too small to make substantive comments on the functional causes of the tumors, and no clear differences between the exposure groups could be inferred using protein function alterations alone. This makes drawing conclusions with respect to translatability from mice to humans difficult. The molecular landscape of the A/J lung tumors studies here (Figure 6) provides an alternative view of the data that may, at least in part, counterbalance these drawbacks. Our prior studies were unable to verify the reported involvement of K-ras mutations in lung tumor progression (Belinsky et al., 1992; Kawano et al., 1996), even though gene expression enrichment analysis suggested activated ras signaling in lung tumors of cigarette smoke-exposed A/J mice, as previously reported (Stinn et al., 2013a). In the current study, and in addition to the classification efforts using the gene signature, we attempted “molecular landscaping” to gain more insights into the make-up of the observed lung tumors. This type of analysis has, to our knowledge, not been done before and highlighted some interesting parallels to lung cancers in smokers. Nevertheless, owing to the limitations of the current study, further independent verifications of our findings are necessary before conclusive statements regarding how appropriate the A/J mouse model is for lung tumorigenesis.

In conclusion, although CS exposure induces tumors in the lungs, air-exposed A/J mice will also develop lung tumors spontaneously as they age. This raises the question whether the CS exposure-induced tumors are of a similar type to spontaneous tumors, irrespective of the overall exposure effect. The challenge is that spontaneous tumors are histologically indistinguishable from the tumors occurring in CS-exposed mice. To tackle the above challenge, a 13-gene gene signature was developed based on an independent A/J mouse CS exposure study, following by a one-class classifier development based on the current study. Identifying gene signature in one data set and building classifier in another data set addresses the feature/gene selection bias which is a well-known problem in literature. We used this 1-class classifier to examine the potential differences between tumors developing in exposed vs. unexposed A/J mice. Tumor classification using this gene signature demonstrated a significant dissimilarity between lung tumors from 3R4F CS-exposed and sham mice. The same signature also highlighted a significant dissimilarity between lung tumors from THS 2.2 aerosol- and 3R4F CS-exposed mice, suggesting a different effect for the 2 exposures. This finding could be confirmed using a similar classification approach with mutational spectra of a subset of the same tumors. Additionally, we provide a unique insight into the molecular landscape of murine lung tumors in the context of this inhalation exposure study. The gene signature was also applied to human lung adenocarcinoma gene expression data (from TCGA) and discriminated cancers in never-smokers from those in ever-smokers, suggesting translatability of our signature genes from mice to humans. This study shows that omics and computational tools provide useful means of tumor classification where histopathological evaluation alone is unsatisfactory to distinguish between age- and exposure-related lung tumors. The results of this study are promising and highlight not only the value of 1-class classifiers when tumor types cannot be easily characterized but also how omics and computational tools can be used to corroborate the relevance of the animal model to exposure effects in humans.

## Data Availability Statement

The datasets presented in this study can be found in online repositories. The names of the repository/repositories and accession number(s) can be found at: https://www.ebi.ac.uk/arrayexpress/, E-MTAB-8540, https://www.ebi.ac.uk/ena, PRJEB34661. Further details on the datasets, the protocols, and additional data visualizations are available on the INTERVALS™ platform at https://doi.org/10.26126/intervals.j3slv2.2.

## Ethics Statement

The animal study was reviewed and approved by the Association for Assessment and Accreditation of Laboratory Animal Care International (AAALAC).

## Author Contributions

YX, FM, JB, and NS performed the computational analysis. KL and EW designed the experiment and interpreted the computational analysis results. All authors contributed to the article and approved the submitted version.

## Conflict of Interest

YX, KL, FM, JB, KT, LN, EW, EG, RD, DP, DB, SO, NS, NI, PV, JH, and MP are employed by Philip Morris International (PMI). AB is an employee of Histovia GmbH, who was contracted and paid by Philip Morris International (PMI).
