# Supplementary material for: Discriminating Spontaneous From Cigarette Smoke and THS 2.2 Aerosol Exposure-Related Proliferative Lung Lesions in A/J Mice by Using Gene Expression and Mutation Spectrum Data
Source: Front Toxicol. 2021 Mar 16;3:634035. doi: 10.3389/ftox.2021.634035 (PMC8915865; doi:10.3389/ftox.2021.634035)
Supplement: Supplementary file 1 [file Data_Sheet_1.docx]

# Discriminating Spontaneous from Cigarette Smoke and THS 2.2 Aerosol Exposure-Related Proliferative Lung Lesions in A/J Mice using Gene Expression and Mutation Spectrum Data

Yang Xiang, Karsta Luettich, Florian Martin, James N. D. Battey, Keyur Trivedi, Laurent Neau, Ee Tsin Wong, Emmanuel Guedj, Remi Dulize, Dariusz Peric, David Bornand, Sonia Ouadi, Nicolas Sierro, Ansgar Büttner, Nikolai V. Ivanov, Patrick Vanscheeuwijck, Julia Hoeng, Manuel C. Peitsch

# Supplementary Material

[Materials and Methods 2](#_Toc45260034)

[Study Design 2](#_Toc45260035)

[Test Atmosphere Generation and Analysis of Test Atmosphere 2](#_Toc45260036)

[Animal Husbandry 4](#_Toc45260037)

[Biomonitoring 5](#_Toc45260038)

[Supplementary Figure 1. Illustration of the interaction analysis 6](#_Toc45260039)

[Supplementary Figure 2. Number of autosomal point mutations in A/J mouse lung tumor samples. 7](#_Toc45260040)

[Supplementary Table 1. Overview of replicate lung samples for analysis. 9](#_Toc45260041)

[Supplementary Table 2. Mahalanobis distances for gene signatures in each individual tumor sample in 3R4F CS- and THS 2.2 aerosol-exposed mice. 10](#_Toc45260042)

[Supplementary Table 3. Mahalanobis distances for mutation spectra in each individual tumor sample in 3R4F CS- and THS 2.2 aerosol-exposed mice. 13](#_Toc45260043)

[Supplementary Table 4. Results of IPA® Core Analysis 15](#_Toc45260044)

# Materials and Methods

## Study Design

A total of 263 male and 990 nulliparous and non-pregnant female A/J mice were randomly allocated to 22 experimental groups on the basis of body weight, sex, dissection time points, and treatment using a Provantis v9.3 (Instem, Staffordshire, UK) randomization sequence. Allocation took into account previously observed mortality rates of 58% and 45% for males and 39% and 20% for females in sham and 3R4F High groups (Stinn et al., 2013), respectively, and the numbers of animals allocated per group for terminal dissection were calculated to ensure at least 50 animals per sex per group at terminal dissection according to OECD Test Guideline 453 (Wong et al., 2020).

Each experimental group contained two to four subgroups which were allocated on the basis of different dissection schemes — histopathology, bronchioalveolar lavage fluid (BALF), lung function, and systems toxicology (genomics, transcriptomics, proteomics). To ensure statistical power for tumor evaluation, the number of animals in the histopathology subgroup at terminal dissection was adjusted, on the basis of historical mortality data (Stinn et al., 2013), to be in accordance with OECD Testing Guideline 453, ensuring a minimum of 50 surviving animals at study termination. For the male mice, the study was terminated after 15 months of exposure to guarantee a minimum of 50 surviving mice at the terminal dissection. The numbers of animals allocated to BALF, lung function, and systems toxicology endpoints were also based on the estimated mortality rates and statistical power calculations to ensure eight to ten animals per group at termination. Animals that were found dead or moribund during the first 6 weeks of the study were replaced with exposed reserve animals belonging to the same group. All exposed female mice were used for analysis of the complete set of endpoints and at four different dissection time points, months 1, 5, 10, and terminal dissection. Male mice were allocated to histopathology and omics endpoints at the terminal dissection time point only. Since female animals are more sensitive to the toxicological effects of CS, assessment of lung inflammation and lung functions at interim dissections (months 1, 5, and 10) was performed in female mice.

## Test Atmosphere Generation and Analysis of Test Atmosphere

Mainstream smoke from 3R4F cigarettes was generated on 30-port rotary smoking machines with 15 ports blocked, with active side stream exhaust (type PMRL-G, SM2000 (Burghart Messtechnik GmbH, Wedel, Germany)) and a programmable dual-port syringe pump. Aerosol from THS 2.2 HeatSticks was generated using a 30-port carousel smoking machine equipped with stick holders and a programmable dual-port syringe pump (designed by PMI and manufactured by Burghart Messtechnik). The THS 2.2 smoking machines also included a temperature-controlled insulation kit (tube warming system) in the undiluted aerosol pathway, with the goal of reducing aerosol condensation. Smoke from 3R4F cigarettes and aerosol from THS 2.2 sticks were generated in accordance with the Health Canada Intense Smoking Protocol (Health Canada, 1999). In addition, several minor deviations from ISO standard 3308 were necessary for technical reasons (Schaller et al., 2016). The THS 2.2 smoking machine was configured to generate 12 puffs per THS 2.2 HeatStick. The 3R4F cigarettes were smoked to average butt lengths of 34.8 ± 0.2 mm and 34.8 ± 0.4 mm, with the resulting average puff count of 10.5 - 10.6 puffs per stick. Two smoking machines were used to produce aerosol to the target nicotine concentration for each of the THS 2.2 (H) chambers. For the 3R4F, THS 2.2 (L) and THS 2.2 (M) groups, one smoking machine each was used to produce the aerosol supplying each of the exposure chambers. The aerosols from the test or reference items were conveyed via glass tubing from the smoking machines to the respective exposure chambers. The comparative analytical characteristics of THS 2.2 aerosol and 3R4F cigarette smoke were previously published (Schaller et al., 2016). For the sham group, filtered, conditioned fresh air was used for exposure.

The test atmosphere in the whole-body exposure chambers was monitored for particle/droplet size distribution and the concentrations of TPM nicotine, carbon monoxide (CO), formaldehyde, acetaldehyde, and acrolein. TPM was gravimetrically (XS 105 DU, Mettler Toledo, Columbus, OH, USA) determined four times per day after trapping particulate matter on a Cambridge-type glass fiber filter pad (Pall Corp, Port Washington, NY, USA). Particle size distribution was determined weekly by using an aerodynamic particle sizer (APS; TSI Inc., Shoreview, MN, USA). The APS was used because mass median aerodynamic diameters (MMAD) and geometric standard deviation (GSD) were internally verified to be equivalent between the APS and the PIXE impactor cascade (Nadaraja et al., 2014). For quantifying nicotine, aerosol was captured four times per day on sulfuric acid-impregnated 3NT EXtrelut^®^ tubes (Merck Millipore, Burlington, MA, USA). Extraction was performed with 5% v/v trimethylamine in *n*-butylacetate (Millipore Sigma, St Louis, MI, USA) prior to analysis by capillary gas chromatography (7890A/7890B series, Agilent Technologies, Santa Clara, CA, USA) with a DB-5 column (Agilent Technologies) by using a flame ionization detector and isoquinoline as internal standard. CO was continuously monitored by nondispersive infrared photometry (Ultramat 6E, Siemens, Brussels, Belgium) of the gas/vapor phase of the test atmospheres. The aldehyde concentrations were determined once per week by reverse-phase high-performance liquid chromatography (HPLC; 1260 series, Agilent Technologies, California, USA) with a Hypersil ODS column (Agilent Technologies) and UV diode array detection of the 2,4-dinitrophenylhydrazine (DNPH (from MilliporeSigma and ITW Reagents, Glenview, IL, USA) derivatives after trapping in acid DNPH (3.23 mM)/acetonitrile solution.

## Animal Husbandry

Animal health status was attested by health check certificates provided by the breeder. Additional health checks comprising comprehensive microbiological, serological, and/or histopathological evaluations were performed by Envigo (Huntingdon, UK) once before start of the exposure period, at 4- to 5-month intervals during the exposure phase, and once at study termination.

The mice were housed under specific hygiene conditions with HEPA-filtered fresh air, at a temperature of 22 ± 3℃ and relative humidity of 55 ± 10%. Positive pressure was maintained inside the animal rooms. The light/dark cycle was 12 h/12 h. Eight to ten mice were housed in each cage. Cage-enrichment, including igloos and nesting paper, were provided to each cage during the non-exposure period. Nesting sheets were provided during the exposure and non-exposure periods. The bedding material (Lignocel^®^ BK 8-15; J. Rettenmaier & Söhne GmbH + Co KG, Rosenberg, Germany) was composed of autoclaved softwood granulate. Bedding material was analyzed by the supplier, and the certificates of analysis were provided to verify that the different batches of bedding material were below the acceptable limits for aflatoxins, selected pesticides, heavy metals, and polychlorinated biphenyls. A gamma-irradiated pellet diet (T2914C irradiated rodent diet; Envigo, Huntingdon, UK) was provided *ad libitum* from the top of the cage through the cage lids. The control (sham and 3R4F CS-exposed groups) and test animals were fed from the same batch of food. The diet was subjected to analysis by the supplier; certificates of analysis were provided to verify the nutritional and chemical profiles of the diet. Additionally, the findings of microbiology tests performed monthly on the diet were within specification. Diet and cage enrichments (except nesting paper) were withdrawn during the exposure period. Filtered tap water in water bottles with steam-sterilized sipper tubes was supplied *ad libitum* for each cage during and in between the exposure periods. The quality of drinking water was monitored by the local monitoring authority to verify adherence to water standards that are based on the World Health Organization Guidelines for Drinking Water (www.pub.gov.sg). Additionally, selected physical (taste and odor), microbiological, and chemical (chloride and sodium) parameters in water were verified to be within specifications by external laboratories on a quarterly basis. Microbiology testing performed monthly in-house was also within the set specification.

During the study, the animals were observed for mortality, clinical signs of toxicity, morbidity, and palpable masses. Mice with identified ailments were tracked daily on a special observation list until they recovered or were removed from the study. Measurement of body weight was performed once per week. Food and water consumption were assessed weekly during the first 13 weeks and once per month thereafter. Food and water consumption were monitored 7 and 3 days per week, respectively. Food consumption data were derived from food that was pooled from all the food consumption cages of the same exposure group to determine the weight difference between the start and end of the food consumption period. Water consumption data were derived from individually weighed water bottles from each cage to determine the weight difference between the start and end of the water consumption period. Scheduled ophthalmoscopy of the experimental animals was performed before the start of the exposure phase (pre-study) and during month 13 of the study. An external examination was performed for the cornea, eyelids, and conjunctiva. The interior of the eyes (lens, corpus vitreous, fundus, and others) was examined by indirect ophthalmoscopy (Keeler All Pupil II, Keeler Ltd, Windsor, UK) after application of a drop of mydriatic eye drops (1% tropicamide, Mydriacyl, Alcon, Fort Worth, TX, USA) approximately 10 min prior to the examination. The animals were momentarily restrained during examination in a quiet and low-illumination environment. A condensing lens of suitable diopter (e.g., 90D, Volk Optical Inc., Mentor, OH, USA) was held approximately 1 cm from the eye during the examination.

## Biomonitoring

Blood was collected from non-anesthetized mice via the facial vein within 10 min post-exposure. Carboxyhemoglobin (CoHb) in heparinized whole blood was monitored up to four times in the study by spectrophotometric measurement by using the COBAS B221 blood gas analyzer (Roche, Basel, Switzerland). Nicotine and cotinine in plasma were monitored four times in the study by Analytisch-biologisches Forschungslabor (ABF) GmbH (Planegg, Germany) by using liquid chromatography–tandem mass spectrometry (LC-MS/MS) (Meger et al., 2002;Scherer et al., 2007a). Urine was collected during the 6-h exposure period by individual placement of animals in an exposure cage with raised bottom grid and during the 18-h post-exposure period by using a urine metabolic cage. The bottom of the exposure cage was rinsed with approximately 100 µL water after exposure, and the solution was pooled with the urine collected from the same animal outside the exposure period, aliquoted, and stored at ≤ −70℃ until analysis. Nicotine metabolites (trans-3′-hydroxycotinine, norcotinine, cotinine, nicotine-*N′*-oxide, and nornicotine) were measured up to four times in the study by HPLC after derivatization with 1,3-diethyl-2-thiobarbituric acid (Millipore Sigma, St Louis, MI, USA) as previously described (Rustemeier et al., 1993). The same samples were submitted for analysis, also performed by ABF GmbH, of other metabolites of HPHCs, including 3-hydroxypropylmercapturic acid (HPMA, metabolite of acrolein), 4-(methylnitrosamino)-1-(3-pyridyl)-1-butanol (NNAL, metabolite of NNK), S-phenylmercapturic acid (SPMA, metabolite of benzene), and 2-cyanoethylmercapturic acid (CEMA, metabolite of acrylonitrile) as described previously (Mascher et al., 2001;Meger et al., 2002;Scherer et al., 2007b;Minet et al., 2011). The results of these analyses are described in another publication (Wong et al., 2020).


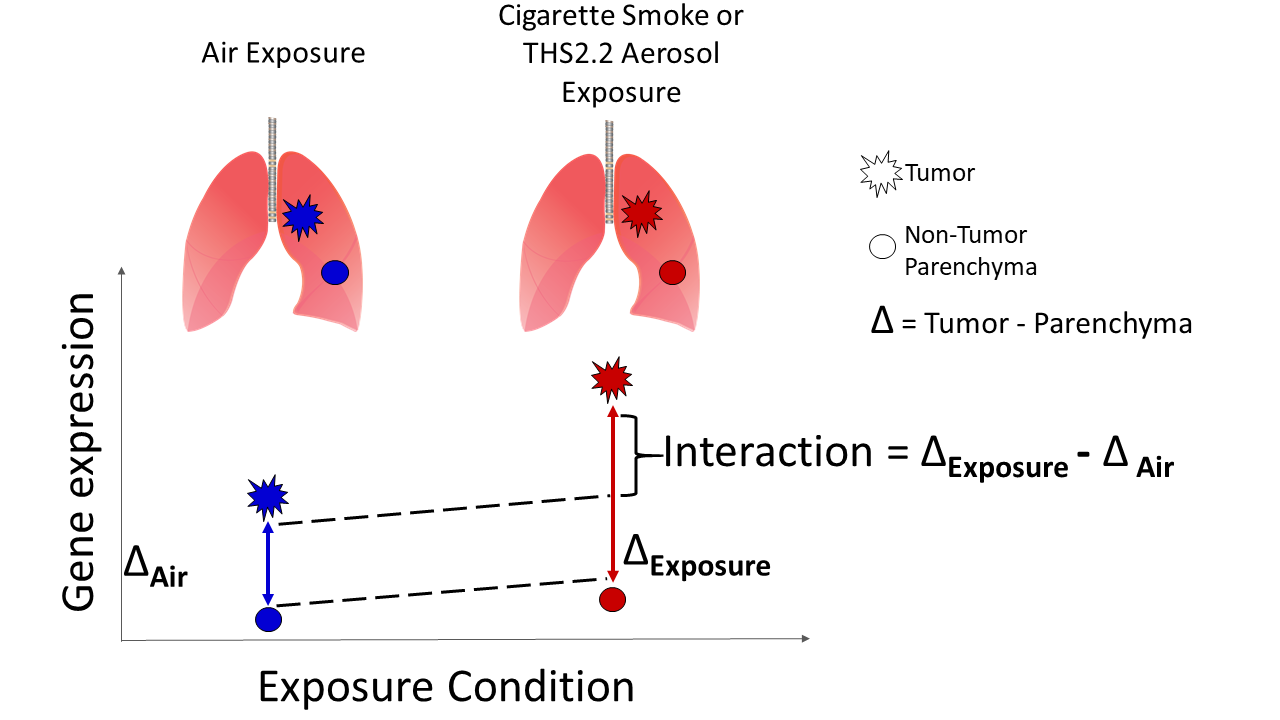


Supplementary Figure 1. Illustration of the interaction analysis

Gene expression responses of two different tissue types—parenchyma (○) and tumor (☼)—in the lungs of air-exposed (Sham) and CS- or THS 2.2 aerosol-exposed mice are illustrated. The interaction term reflects the changes in gene expression, which were different in tumors compared to the surrounding parenchyma tissues following exposure. In other words, the genes with significant interaction are those whose levels were differentially affected between the two tissue types upon exposure.


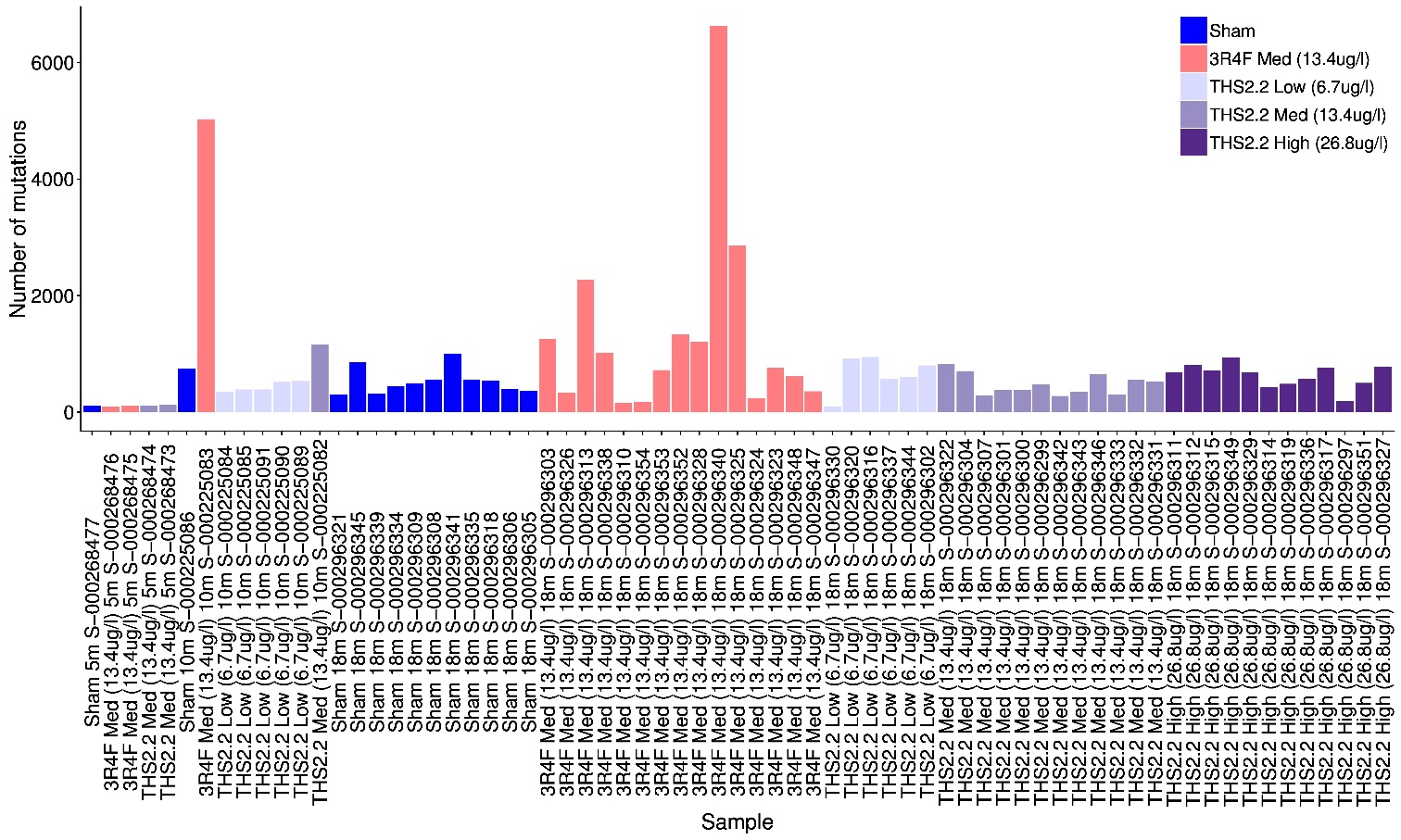


Supplementary Figure 2. Number of autosomal point mutations in A/J mouse lung tumor samples.

The number of mutations (on the *y*-axis) is plotted as a bar for each tumor sample (on the *x*-axis).

Supplementary Figure 3. Relative proportion of the six mutation types in A/J mouse lung tumors.

Proportions of each of the six mutation types (C→A, C→G, C→T, T→A, T→C, T→G, indicated by different colors, see legend) for each tumor sample are shown as stacked bar chart. Tumor samples are indicated by their study group designation and sample ID listed on the right side. The dendrogram on the left reflects the clustering of the samples based on the relative proportion of each base substitution type displayed in the bar chart.


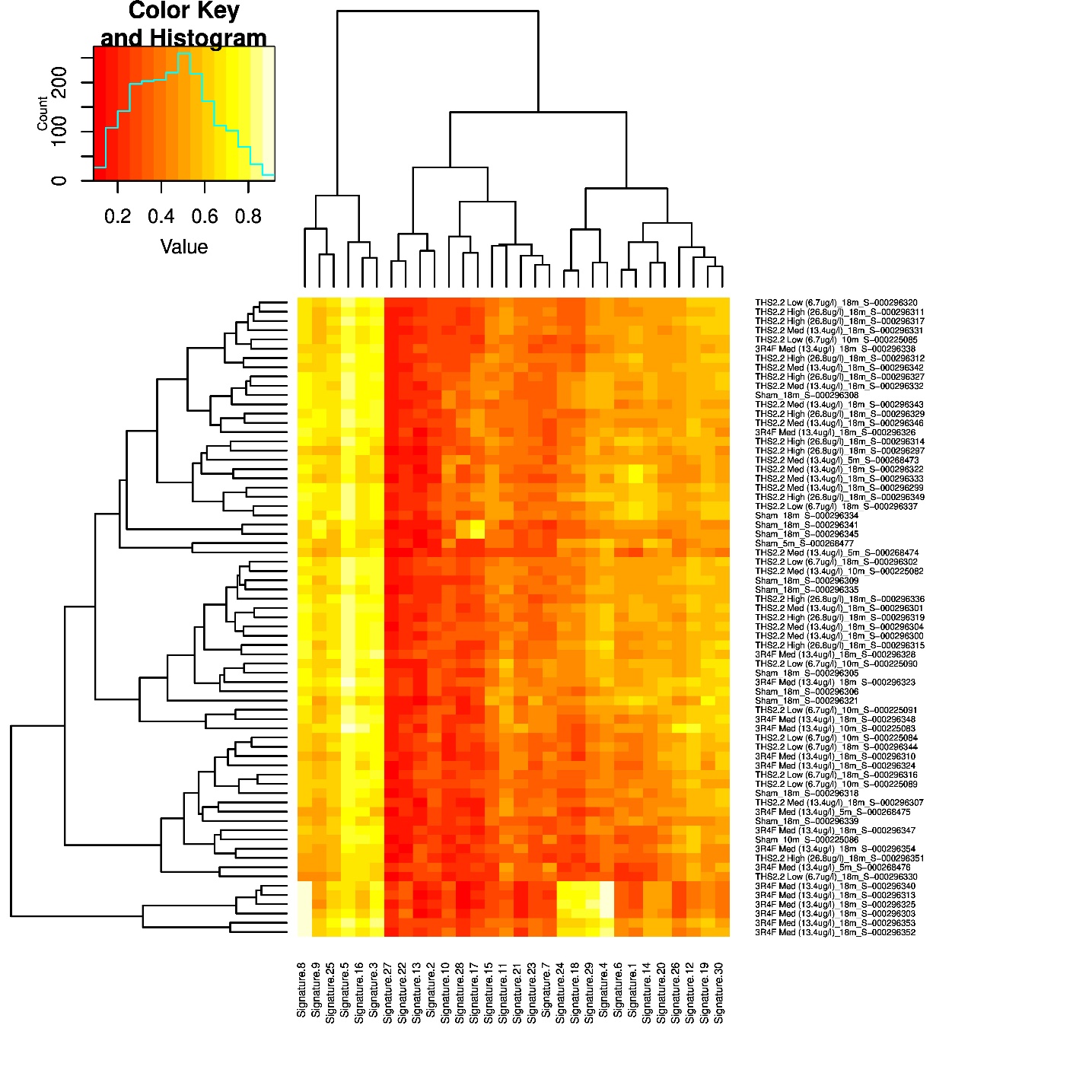


**Supplementary Figure 4. Cosine similarities between the mutational profiles of the tumor samples and the profiles in the COSMIC mutation signature database.**

Mutational signature similarities were calculated using the Mutational Patterns library in R (Blokzijl et al., 2018), using the Cosmic v2 mutational signatures (Alexandrov, 2015). A group of 18 month 3R4F exposure samples group closely together (bottom 5 rows) and apart from the other samples. The separation is driven by multiple signatures, including signature 4, which is commonly associated with, amongst others, lung cancer, and whose proposed etiology is tobacco smoking (Alexandrov, 2015).

Supplementary Table 1. Overview of replicate lung samples for gene expression analysis. The sham group was also called non-exposed group or air-exposed group. There were three types of lung tumors: i) spontaneous tumors, which developed spontaneously in air-exposed A/J mice with age; ii) 3R4F CS-related tumors, which occurred in CS-exposed mice; iii) tumors from THS 2.2 aerosol-exposed mice.

| **Study Group Designation** |  | **Sample Type** | **No. of LCM Samples for Gene Expression Analysis** | **Number of QC-passed CEL Files** |
| --- | --- | --- | --- | --- |
| Sham 5m F |  | Parenchyma | 8 | 7 |
|  |  | spontaneous Tumor | 1 | 0 |
| 3R4F Med (13.4µg/l) 5m F |  | Parenchyma | 9* | 9* |
|  |  | CS-related Tumor | 3 | 3 |
| THS2.2 Low (6.7µg/l) 5m F |  | Parenchyma | 8 | 8 |
|  |  | Tumor from THS 2.2 aerosol-exposed mice | 0 | 0 |
| THS2.2 Med (13.4µg/l) 5m F |  | Parenchyma | 8 | 7 |
|  |  | Tumor from THS 2.2 aerosol-exposed mice | 2 | 2 |
| THS2.2 High (26.8µg/l) 5m F |  | Parenchyma | 8 | 8 |
|  |  | Tumor from THS 2.2 aerosol-exposed mice | 1 | 1 |
| Sham 10m F |  | Parenchyma | 10 | 10 |
|  |  | spontaneous Tumor | 5 | 4 |
| 3R4F Med (13.4µg/l) 10m F |  | Parenchyma | 12 | 7 |
|  |  | CS-related Tumor | 10 | 5 |
| THS2.2 Low (6.7µg/l) 10m F |  | Parenchyma | 12 | 12 |
|  |  | Tumor from THS 2.2 aerosol-exposed mice | 5 | 5 |
| THS2.2 Med (13.4µg/l) 10m F |  | Parenchyma | 11 | 11 |
|  |  | Tumor from THS 2.2 aerosol-exposed mice | 4 | 4 |
| THS2.2 High (26.8µg/l) 10m F |  | Parenchyma | 12 | 12 |
|  |  | Tumor from THS 2.2 aerosol-exposed mice | 1 | 0 |
| Sham 18m F |  | Parenchyma | 10 | 10 |
|  |  | spontaneous Tumor | 5 | 5 |
| 3R4F Med (13.4µg/l) 18m F |  | Parenchyma | 10 | 10 |
|  |  | CS-related Tumor | 20 | 16 |
| THS2.2 Low (6.7µg/l) 18m F |  | Parenchyma | 10 | 10 |
|  |  | Tumor from THS 2.2 aerosol-exposed mice | 9 | 9 |
| THS2.2 Med (13.4µg/l) 18m F |  | Parenchyma | 13 | 13 |
|  |  | Tumor from THS 2.2 aerosol-exposed mice | 15 | 12 |
| THS2.2 High (26.8µg/l) 18m F |  | Parenchyma | 12 | 12 |
|  |  | Tumor from THS 2.2 aerosol-exposed mice | 10 | 9 |
| Sham 15m M |  | Parenchyma | 16 | 16 |
|  |  | spontaneous Tumor | 8 | 8 |
| THS2.2 High (28.6µg/l) 15m M |  | Parenchyma | 5 | 5 |
|  |  | Tumor from THS 2.2 aerosol-exposed mice | 2 | 2 |

*) Two parenchyma samples from animal 2052033 were processed and analyzed.

Supplementary Table 2. Mahalanobis distances for gene signatures in each individual tumor sample in 3R4F CS- and THS 2.2 aerosol-exposed mice.

| **Sample ID** | **Study Group Designation** | **Distance from the center of sham group** |
| --- | --- | --- |
| S-000151895 | THS2.2 6.7 ug nicotine/L 10m female | 12.36646 |
| S-000151903 | Sham 10m female | 3.044643 |
| S-000151906 | THS2.2 13.4 ug nicotine/L 10m female | 16.90286 |
| S-000151907 | THS2.2 6.7 ug nicotine/L 10m female | 2.497955 |
| S-000151909 | Sham 10m female | 6.29533 |
| S-000151912 | THS2.2 6.7 ug nicotine/L 10m female | 17.66716 |
| S-000151913 | Sham 10m female | 10.53108 |
| S-000151914 | Sham 10m female | 3.395593 |
| S-000151915 | THS2.2 6.7 ug nicotine/L 10m female | 6.414093 |
| S-000151916 | 3R4F 10m female | 40.43582 |
| S-000151917 | 3R4F 10m female | 35.73043 |
| S-000151918 | 3R4F 10m female | 44.1788 |
| S-000257797 | THS2.2 26.8 ug nicotine/L 05m female | 23.93633 |
| S-000257798 | THS2.2 13.4 ug nicotine/L 05m female | 8.734665 |
| S-000257799 | THS2.2 13.4 ug nicotine/L 05m female | 6.4664 |
| S-000257800 | 3R4F 05m female | 50.82365 |
| S-000257801 | 3R4F 05m female | 21.83027 |
| S-000257802 | 3R4F 05m female | 28.39662 |
| S-000296064 | THS2.2 6.7 ug nicotine/L 18m female | 2.418845 |
| S-000296066 | 3R4F 18m female | 10.85543 |
| S-000296067 | 3R4F 18m female | 22.37808 |
| S-000296068 | THS2.2 13.4 ug nicotine/L 18m female | 8.938884 |
| S-000296069 | THS2.2 13.4 ug nicotine/L 18m female | 14.32292 |
| S-000296072 | THS2.2 6.7 ug nicotine/L 18m female | 11.55814 |
| S-000296073 | Sham 18m female | 3.508819 |
| S-000296074 | THS2.2 26.8 ug nicotine/L 18m female | 9.47005 |
| S-000296076 | Sham 18m male | 5.281386 |
| S-000296077 | THS2.2 26.8 ug nicotine/L 18m male | 19.37262 |
| S-000296078 | 3R4F 18m female | 16.10544 |
| S-000296079 | THS2.2 26.8 ug nicotine/L 18m female | 31.21493 |
| S-000296080 | Sham 18m male | 6.143442 |
| S-000296081 | Sham 18m male | 9.394874 |
| S-000296082 | 3R4F 18m female | 17.83662 |
| S-000296084 | 3R4F 18m female | 31.69619 |
| S-000296085 | THS2.2 6.7 ug nicotine/L 18m female | 3.557942 |
| S-000296086 | THS2.2 26.8 ug nicotine/L 18m male | 14.86169 |
| S-000296087 | THS2.2 6.7 ug nicotine/L 18m female | 14.85667 |
| S-000296088 | THS2.2 26.8 ug nicotine/L 18m female | 7.444695 |
| S-000296089 | THS2.2 26.8 ug nicotine/L 18m female | 11.75956 |
| S-000296090 | Sham 18m female | 27.53426 |
| S-000296092 | THS2.2 13.4 ug nicotine/L 18m female | 8.561945 |
| S-000296093 | THS2.2 13.4 ug nicotine/L 18m female | 13.05986 |
| S-000296094 | THS2.2 13.4 ug nicotine/L 18m female | 17.06257 |
| S-000296095 | THS2.2 13.4 ug nicotine/L 18m female | 16.15469 |
| S-000296096 | 3R4F 18m female | 33.56536 |
| S-000296097 | 3R4F 18m female | 15.3034 |
| S-000296099 | THS2.2 13.4 ug nicotine/L 18m female | 1.676677 |
| S-000296100 | THS2.2 13.4 ug nicotine/L 18m female | 12.26139 |
| S-000296101 | THS2.2 6.7 ug nicotine/L 18m female | 9.76413 |
| S-000296102 | Sham 18m female | 8.582279 |
| S-000296103 | THS2.2 26.8 ug nicotine/L 18m female | 5.413164 |
| S-000296104 | 3R4F 18m female | 32.55926 |
| S-000296105 | THS2.2 26.8 ug nicotine/L 18m female | 10.79465 |
| S-000296106 | THS2.2 6.7 ug nicotine/L 18m female | 11.71468 |
| S-000296107 | THS2.2 6.7 ug nicotine/L 18m female | 4.711046 |
| S-000296108 | THS2.2 13.4 ug nicotine/L 18m female | 5.168752 |
| S-000296110 | Sham 18m male | 2.692201 |
| S-000296111 | Sham 18m male | 50.1805 |
| S-000296117 | THS2.2 26.8 ug nicotine/L 18m female | 25.18426 |
| S-000296118 | Sham 18m female | 4.892314 |
| S-000296119 | 3R4F 18m female | 5.738296 |
| S-000296120 | 3R4F 18m female | 7.103885 |
| S-000296121 | 3R4F 18m female | 33.29251 |
| S-000296122 | 3R4F 18m female | 11.23546 |
| S-000296123 | THS2.2 26.8 ug nicotine/L 18m female | 18.40551 |
| S-000296124 | THS2.2 13.4 ug nicotine/L 18m female | 9.72896 |
| S-000296125 | 3R4F 18m female | 12.0586 |
| S-000296126 | THS2.2 13.4 ug nicotine/L 18m female | 5.192193 |
| S-000296127 | Sham 18m male | 2.775434 |
| S-000296128 | Sham 18m male | 3.543063 |
| S-000296129 | THS2.2 26.8 ug nicotine/L 18m female | 8.752667 |
| S-000296130 | THS2.2 6.7 ug nicotine/L 18m female | 15.56356 |
| S-000296131 | THS2.2 6.7 ug nicotine/L 18m female | 7.146998 |
| S-000296132 | Sham 18m female | 4.819787 |
| S-000151894 | THS2.2 6.7 ug nicotine/L 10m female | 14.24399 |
| S-000296091 | Sham 18m male | 8.162177 |
| S-000296098 | THS2.2 13.4 ug nicotine/L 18m female | 8.041996 |
| S-000151898 | 3R4F 10m female | 16.68136 |
| S-000151900 | 3R4F 10m female | 17.55039 |
| S-000151901 | THS2.2 13.4 ug nicotine/L 10m female | 12.99861 |
| S-000151902 | THS2.2 13.4 ug nicotine/L 10m female | 7.572659 |
| S-000151911 | THS2.2 13.4 ug nicotine/L 10m female | 15.19817 |
| S-000296112 | 3R4F 18m female | 30.30572 |
| S-000296114 | 3R4F 18m female | 21.18025 |
| S-000296115 | 3R4F 18m female | 53.37922 |

Supplementary Table 3. Mahalanobis distances for mutation spectra in each individual tumor sample in 3R4F CS- and THS 2.2 aerosol-exposed mice.

| **Sample ID** | **Study Group Designation** | **Distance from the center of sham group** |
| --- | --- | --- |
| S-000151894 | THS2.2 6.7 ug nicotine/L 10m female | 8.554821663 |
| S-000151895 | THS2.2 6.7 ug nicotine/L 10m female | 29.86281862 |
| S-000151906 | THS2.2 13.4 ug nicotine/L 10m female | 2.083088324 |
| S-000151907 | THS2.2 6.7 ug nicotine/L 10m female | 3.70835341 |
| S-000151912 | THS2.2 6.7 ug nicotine/L 10m female | 1.991018134 |
| S-000151914 | Sham 10m female | 6.039231658 |
| S-000151915 | THS2.2 6.7 ug nicotine/L 10m female | 6.308284279 |
| S-000296064 | THS2.2 6.7 ug nicotine/L 18m female | 8.970246 |
| S-000296066 | 3R4F 18m female | 96.93647988 |
| S-000296067 | 3R4F 18m female | 0.879075877 |
| S-000296068 | THS2.2 13.4 ug nicotine/L 18m female | 6.397216742 |
| S-000296069 | THS2.2 13.4 ug nicotine/L 10m female | 1.646236118 |
| S-000296072 | THS2.2 6.7 ug nicotine/L 18m female | 8.679080115 |
| S-000296073 | Sham 18m female | 8.420595858 |
| S-000296074 | THS2.2 26.8 ug nicotine/L 18m female | 0.674124273 |
| S-000296076 | Sham 15m male | 4.714259779 |
| S-000296077 | THS2.2 26.8 ug nicotine/L 15m male | 17.69905383 |
| S-000296078 | 3R4F 18m female | 6.302885885 |
| S-000296079 | THS2.2 26.8 ug nicotine/L 18m female | 4.645123825 |
| S-000296080 | Sham 18m male | 3.684342937 |
| S-000296081 | Sham 15m male | 4.51925292 |
| S-000296082 | 3R4F 18m female | 22.06987068 |
| S-000296084 | 3R4F 18m female | 1.64834996 |
| S-000296086 | THS2.2 26.8 ug nicotine/L 15m male | 3.946259259 |
| S-000296087 | THS2.2 6.7 ug nicotine/L 18m female | 1.148922875 |
| S-000296088 | THS2.2 26.8 ug nicotine/L 18m female | 1.779479187 |
| S-000296089 | THS2.2 26.8 ug nicotine/L 18m female | 23.77638183 |
| S-000296090 | Sham 18m female | 5.443626406 |
| S-000296091 | Sham 15m male | 2.80836579 |
| S-000296092 | THS2.2 13.4 ug nicotine/L 18m female | 7.447182048 |
| S-000296093 | THS2.2 13.4 ug nicotine/L 18m female | 3.623634055 |
| S-000296095 | THS2.2 13.4 ug nicotine/L 18m female | 3.561581282 |
| S-000296097 | 3R4F 18m female | 31.9467291 |
| S-000296098 | THS2.2 13.4 ug nicotine/L 18m female | 1.598568262 |
| S-000296099 | THS2.2 13.4 ug nicotine/L 18m female | 2.612960142 |
| S-000296100 | THS2.2 13.4 ug nicotine/L 18m female | 5.415916266 |
| S-000296101 | THS2.2 6.7 ug nicotine/L 18m female | 4.280911353 |
| S-000296102 | Sham 18m female | 1.310978255 |
| S-000296103 | THS2.2 26.8 ug nicotine/L 18m female | 5.247918523 |
| S-000296104 | 3R4F 18m female | 144.8885903 |
| S-000296105 | THS2.2 26.8 ug nicotine/L 18m female | 4.853086857 |
| S-000296106 | THS2.2 6.7 ug nicotine/L 18m female | 12.45682582 |
| S-000296107 | THS2.2 6.7 ug nicotine/L 18m female | 0.803014879 |
| S-000296110 | Sham 15m male | 3.404921374 |
| S-000296112 | 3R4F 18m female | 105.7094428 |
| S-000296114 | 3R4F 18m female | 154.7485428 |
| S-000296117 | THS2.2 26.8 ug nicotine/L 18m female | 18.65920971 |
| S-000296119 | 3R4F 18m female | 25.06156426 |
| S-000296121 | 3R4F 18m female | 35.03707787 |
| S-000296122 | 3R4F 18m female | 13.43786491 |
| S-000296123 | THS2.2 26.8 ug nicotine/L 18m female | 1.545817995 |
| S-000296124 | THS2.2 13.4 ug nicotine/L 18m female | 3.308201297 |
| S-000296125 | 3R4F 18m female | 14.69901542 |
| S-000296126 | THS2.2 13.4 ug nicotine/L 18m female | 2.925192022 |
| S-000296127 | Sham 15m male | 1.409540315 |
| S-000296128 | Sham 15m male | 6.58150385 |
| S-000296129 | THS2.2 26.8 ug nicotine/L 18m female | 9.23213985 |
| S-000296132 | Sham 18m female | 6.523861349 |

Supplementary Table 4. Results of IPA® Core Analysis

Provided as separate .xlsx file

Supplementary Table 5. Overview of replicate lung samples for mutation analysis.

**Animals**=number of animals from which samples were taken.

**Received**=the number of samples received.

**Libraries attempted**=number of samples for which DNA isolation and library prep was attempted.

**Libraries Sequenced**=the number of samples which were sent for sequencing.

**Analyzed**=the number of samples successfully sent for mutation calling (groups from single animals, with at least one tumor and at least one parenchyma).

**Combined**=the number of analyzed samples that could be combined with the transcriptomics samples (COG).

**NA**=Not applicable.

| **Condition** | **Sample Type** | **Animals** | **Received** | **Libraries attempted** | **Libraries sequenced** | **Analyzed** | **Combined** |
| --- | --- | --- | --- | --- | --- | --- | --- |
| Sham 5m F | Parenchyma | 7 | 14 | 14 | 14 | 2 | NA |
| Sham 5m F | Tumor | 1 | 1 | 1 | 1 | 1 | NA |
| 3R4F Med (13.4ug/l) 5m F | Parenchyma | 8 | 16 | 16 | 16 | 2 | NA |
| 3R4F Med (13.4ug/l) 5m F | Tumor | 1 | 3 | 2 | 2 | 2 | NA |
| THS2.2 Low (6.7ug/l) 5m F | Parenchyma | 8 | 16 | 16 | 15 | 0 | NA |
| THS2.2 Low (6.7ug/l) 5m F | Tumor | 0 | 0 | 0 | 0 | 0 | NA |
| THS2.2 Med (13.4ug/l) 5m F | Parenchyma | 8 | 16 | 16 | 16 | 4 | NA |
| THS2.2 Med (13.4ug/l) 5m F | Tumor | 2 | 2 | 2 | 2 | 2 | NA |
| THS2.2 High (26.8ug/l) 5m F | Parenchyma | 8 | 16 | 16 | 16 | 0 | NA |
| THS2.2 High (26.8ug/l) 5m F | Tumor | 1 | 1 | 0 | 0 | 0 | NA |
| Sham 10m F | Parenchyma | 10 | 10 | 10 | 10 | 1 | NA |
| Sham 10m F | Tumor | 1 | 1 | 1 | 1 | 1 | 1 |
| 3R4F Med (13.4ug/l) 10m F | Parenchyma | 12 | 12 | 11 | 11 | 1 | NA |
| 3R4F Med (13.4ug/l) 10m F | Tumor | 2 | 2 | 2 | 1 | 1 | 0 |
| THS2.2 Low (6.7ug/l) 10m F | Parenchyma | 12 | 12 | 12 | 12 | 4 | NA |
| THS2.2 Low (6.7ug/l) 10m F | Tumor | 4 | 5 | 5 | 5 | 5 | 5 |
| THS2.2 Med (13.4ug/l) 10m F | Parenchyma | 11 | 12 | 11 | 11 | 1 | NA |
| THS2.2 Med (13.4ug/l) 10m F | Tumor | 1 | 1 | 1 | 1 | 1 | 1 |
| THS2.2 High (26.8ug/l) 10m F | Parenchyma | 12 | 12 | 11 | 11 | 0 | NA |
| THS2.2 High (26.8ug/l) 10m F | Tumor | 1 | 1 | 1 | 1 | 0 | 0 |
| Sham 18m F | Parenchyma | 10 | 20 | 20 | 10 | 4 | NA |
| Sham 18m F | Tumor | 4 | 4 | 4 | 4 | 4 | 4 |
| 3R4F Med (13.4ug/l) 18m F | Parenchyma | 10 | 20 | 20 | 10 | 8 | NA |
| 3R4F Med (13.4ug/l) 18m F | Tumor | 8 | 15 | 15 | 15 | 15 | 13 |
| THS2.2 Low (6.7ug/l) 18m F | Parenchyma | 10 | 20 | 20 | 10 | 6 | NA |
| THS2.2 Low (6.7ug/l) 18m F | Tumor | 7 | 8 | 8 | 8 | 6 | 6 |
| THS2.2 Med (13.4ug/l) 18m F | Parenchyma | 13 | 26 | 26 | 13 | 8 | NA |
| THS2.2 Med (13.4ug/l) 18m F | Tumor | 8 | 12 | 12 | 12 | 12 | 10 |
| THS2.2 High (26.8ug/l) 18m F | Parenchyma | 12 | 24 | 24 | 12 | 9 | NA |
| THS2.2 High (26.8ug/l) 18m F | Tumor | 9 | 10 | 10 | 10 | 10 | 9 |
| Sham 15m M | Parenchyma | 16 | 32 | 32 | 16 | 5 | NA |
| Sham 15m M | Tumor | 5 | 7 | 7 | 7 | 7 | 7 |
| THS2.2 High (26.8ug/l) 15m M | Parenchyma | 5 | 10 | 10 | 5 | 2 | NA |
| THS2.2 High (26.8ug/l) 15m M | Tumor | 2 | 2 | 2 | 2 | 2 | 2 |
| Column totals | NA | 229 | 363 | 358 | 280 | 126 | 58 |

Alexandrov, L.B. (2015). Understanding the origins of human cancer. *Science* 350**,** 1175.

Blokzijl, F., Janssen, R., Van Boxtel, R., and Cuppen, E. (2018). MutationalPatterns: comprehensive genome-wide analysis of mutational processes. *Genome Med* 10**,** 33.

Health Canada (1999). "Health Canada T-115: Determination of "Tar", Nicotine and Carbon Monoxide in Mainstream Tobacco Smoke".).

Mascher, D.G., Mascher, H.J., Scherer, G., and Schmid, E.R. (2001). High-performance liquid chromatographic-tandem mass spectrometric determination of 3-hydroxypropylmercapturic acid in human urine. *J Chromatogr B Biomed Sci Appl* 750**,** 163-169.

Meger, M., Meger-Kossien, I., Schuler-Metz, A., Janket, D., and Scherer, G. (2002). Simultaneous determination of nicotine and eight nicotine metabolites in urine of smokers using liquid chromatography-tandem mass spectrometry. *J Chromatogr B Analyt Technol Biomed Life Sci* 778**,** 251-261.

Minet, E., Cheung, F., Errington, G., Sterz, K., and Scherer, G. (2011). Urinary excretion of the acrylonitrile metabolite 2-cyanoethylmercapturic acid is correlated with a variety of biomarkers of tobacco smoke exposure and consumption. *Biomarkers* 16**,** 89-96.

Nadaraja, N., Joseph, T., Valdez, F., and Tan, W.T. (2014). Aerodynamic particle sizer (APS) 4571. *PMIRL Singapore Process Verification*.

Rustemeier, K., Demetriou, D., Schepers, G., and Voncken, P. (1993). High-performance liquid chromatographic determination of nicotine and its urinary metabolites via their 1,3-diethyl-2-thiobarbituric acid derivatives. *J Chromatogr* 613**,** 95-103.

Schaller, J.P., Keller, D., Poget, L., Pratte, P., Kaelin, E., Mchugh, D., Cudazzo, G., Smart, D., Tricker, A.R., Gautier, L., Yerly, M., Reis Pires, R., Le Bouhellec, S., Ghosh, D., Hofer, I., Garcia, E., Vanscheeuwijck, P., and Maeder, S. (2016). Evaluation of the Tobacco Heating System 2.2. Part 2: Chemical composition, genotoxicity, cytotoxicity, and physical properties of the aerosol. *Regul Toxicol Pharmacol* 81 Suppl 2**,** S27-S47.

Scherer, G., Engl, J., Urban, M., Gilch, G., Janket, D., and Riedel, K. (2007a). Relationship between machine-derived smoke yields and biomarkers in cigarette smokers in Germany. *Regul Toxicol Pharmacol* 47**,** 171-183.

Scherer, G., Urban, M., Hagedorn, H.W., Feng, S., Kinser, R.D., Sarkar, M., Liang, Q., and Roethig, H.J. (2007b). Determination of two mercapturic acids related to crotonaldehyde in human urine: influence of smoking. *Hum Exp Toxicol* 26**,** 37-47.

Stinn, W., Berges, A., Meurrens, K., Buettner, A., Gebel, S., Lichtner, R.B., Janssens, K., Veljkovic, E., Xiang, Y., Roemer, E., and Haussmann, H.J. (2013). Towards the validation of a lung tumorigenesis model with mainstream cigarette smoke inhalation using the A/J mouse. *Toxicology* 305**,** 49-64.

Wong, E.T., Luettich, K., Krishnan, S., Wong, S.K., Lim, W.T., Yeo, D., Büttner, A., Leroy, P., Vuillaume, G., Boué, S., Hoeng, J., Vanscheeuwijck, P., and Peitsch, M.C. (2020). Reduced Chronic Toxicity and Carcinogenicity in A/J Mice in Response to Life-Time Exposure to Aerosol from a Heated Tobacco Product Compared with Cigarette Smoke. *Toxicological Sciences* Accepted for publication.
